# Evolutionary History of the Photolyase/Cryptochrome Superfamily in Eukaryotes

**DOI:** 10.1371/journal.pone.0135940

**Published:** 2015-09-09

**Authors:** Qiming Mei, Volodymyr Dvornyk

**Affiliations:** 1 Key Laboratory of Vegetation Restoration and Management of Degraded Ecosystems, South China Botanical Garden, Chinese Academy of Sciences, Guangzhou, People’s Republic of China; 2 School of Biological Sciences, the University of Hong Kong, Pokfulam Rd., Hong Kong SAR, People’s Republic of China; 3 Department of Life Sciences, College of Science and General Studies, Alfaisal University, Riyadh, Kingdom of Saudi Arabia; Karlsruhe Institute of Technology, GERMANY

## Abstract

**Background:**

Photolyases and cryptochromes are evolutionarily related flavoproteins, which however perform distinct physiological functions. Photolyases (PHR) are evolutionarily ancient enzymes. They are activated by light and repair DNA damage caused by UV radiation. Although cryptochromes share structural similarity with DNA photolyases, they lack DNA repair activity. Cryptochrome (CRY) is one of the key elements of the circadian system in animals. In plants, CRY acts as a blue light receptor to entrain circadian rhythms, and mediates a variety of light responses, such as the regulation of flowering and seedling growth.

**Results:**

We performed a comprehensive evolutionary analysis of the CRY/PHR superfamily. The superfamily consists of 7 major subfamilies: CPD class I and CPD class II photolyases, (6–4) photolyases, CRY-DASH, plant PHR2, plant CRY and animal CRY. Although the whole superfamily evolved primarily under strong purifying selection (average ω = 0.0168), some subfamilies did experience strong episodic positive selection during their evolution. Photolyases were lost in higher animals that suggests natural selection apparently became weaker in the late stage of evolutionary history. The evolutionary time estimates suggested that plant and animal CRYs evolved in the Neoproterozoic Era (~1000–541 Mya), which might be a result of adaptation to the major climate and global light regime changes occurred in that period of the Earth’s geological history.

## Introduction

Photolyases are light-dependent DNA repair enzymes. They are activated by blue light and repair UV induced DNA damage by removing pyrimidine dimers. Three types of PHRs have been identified: CPD photolyases repair cyclobutane pyrimidine dimers, (6–4) photolyases repair (6–4) pyrimidine pyrimidone, and cryptochrome-DASHs exhibit a variety of physiological functions including single-strand DNA photolyase activity [1,2], transcriptional regulation in *Synechocystis* [3] and light-dependent regulation of metabolism in *Fusarium* [4].

Photolyases are evolutionary old proteins found in many species from bacteria to vertebrates [1,5]. Recent studies suggested that DNA repair might have a common evolutionary origin with circadian rhythmicity [6]. Circadian rhythmicity is a roughly 24-hour cycle of biochemical, physiological, and behavioral processes. It was found in both prokaryotes and eukaryotes [7]. Circadian rhythms enhance fitness of organisms in both constant and changing environments [8]. A circadian clock system consists of a central oscillator, input and output pathways [9]. The central oscillator is able to maintain the rhythmic output in the absence of the external stimuli [10]. Unicellular organisms rely on a single independent circadian oscillator, whereas organisms with differentiated tissues may have multi-oscillator systems to coordinate with different rhythms [7]. In animals, the central oscillator resides in the brain, which controls the circadian behavior of the whole organism and synchronizes peripheral clocks in other organs [11].

The circadian oscillators of eukaryotes have been extensively studied in fruit fly and mammals [12–14]. One of the key elements of the circadian system in animals and plants is CRY; in plants it acts as a blue light receptor to entrain circadian rhythms [15].

**Fig 1:**
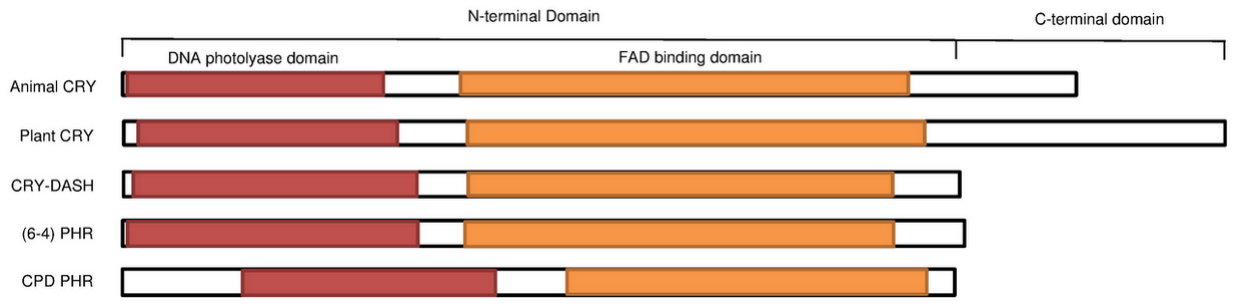
Domain architecture of the CRY/PHR superfamily. The conserved domains were identified by the Conserved Domains Database (CDD) tool [19]. The reference sequences are as follows. Animal CRY: *Homo sapiens* CRY1 (NP_004066); plant CRY: *Arabidopsis thaliana* CRY1 (NP_567341); CRY-DASH: *Xenopus laevis* (NP_001084438); (6–4) PHR: *Danio rerio* (NP_571863); CPD PHR: *D*. *rerio* CPD class II PHR (NP_957358). The DNA photolyase related domain and a FAD binding domain are shared by both CRY and PHR; whereas the C-terminal variable domain is only present in CRY but not in PHY.

Cryptochromes are flavoproteins, which are homologous to photolyases but lack the DNA repair activity [16]. Cryptochromes and photolyases form the photolyase/cryptochrome superfamily [17]. Cryptochromes are ubiquitous in plants and animals [11]. Photolyases and cryptochromes have two conserved domains, a DNA photolyase related domain and a FAD binding domain. In addition, plant and animal cryptochromes possess a C-terminal domain of a variable length, which is absent in the CRY-DASH and photolyase proteins [18] (Fig 1, the conserved domains were identified by the Conserved Domains Database (CDD) tool [19]). The variation in the length of the C-terminal domains results in functional diversity within the cryptochrome family [20]. The C-terminus of the mammalian cryptochrome possesses a nuclear localization domain required for CRY’s nuclear localization; deletion of the C-terminus prevents mammalian CRY from negatively regulating the transcription of other circadian components. The C-terminal domain of the *Arabidopsis* CRY is essential for mediating the signaling mechanism by responding to the light [21].

Cryptochromes possess two chromophores: pterin (in the form of 5, 10-methenyltetrahydrofolate, MTHF) and flavin (in the form of flavin adenine dinucleotide, FAD); they bind to the DNA photolyase related region and FAD binding domain respectively as cofactors that absorb light [22]. Photolyases also have two cofactors, one of them is FAD, and another can be either 5,10-methenyl-tetrahydrofolate (MTHF) or 8-hydroxy-5-deazaflavin (8-HDF) [22]. The 3D-architectures of the photolyase/cryptochrome superfamily members are similar. All of them fold into 2 domains, an α/β domain and a helical domain. These 2 domains are connected by a variable loop and the 2 lobes of the helical domain form a groove, which is called FAD-access cavity. FAD embeds in this “molecular pocket”, and may be resolved at the bottom [23].

Studies on molecular evolution of the CRY/PHR superfamily are somewhat limited. The pioneering study of Kanai et al. [24] was conducted on the relatively small number of sequences and thus lacked generalization. Lucas-Lledo and Lynch [5] performed more comprehensive phylogenomic analysis of photolyases focused primarily at the gene gain-loss events and mutation rates. Our study presents the results of the much more comprehensive analysis of the occurrence, phylogeny, selection, conservation and evolutionary time scale of the photolyase superfamily in eukaryotes.

## Materials and Methods

### DNA and protein sequences

DNA and protein sequences of CRY and PHR were retrieved from the GenBank; only the sequences from fully sequenced eukaryotic genomes were used for the analyses. We used BLASTP and TBLASTN [25] to search the protein database. The following protein sequences were selected as the queries for respective groups of organisms: *Homo sapiens* (NP_004066 and NP_066940) and *Aedes aegypti* (XP_001648498 and XP_001655778) for animal CRY; *Xenopus laevis* (NP_001081421), *Drosophila melanogaster* (NP_724274), *Monosiga brevicollis* (XP_001747506), *Arabidopsis thaliana* (NP_566520) and *Verticillium albo-atrum* (XP_003006428) for (6–4) PHR; *Salpingoeca* sp. ATCC 50818 (XP_004989008), *Arabidopsis thaliana* (NP_568461) and *Verticillium albo-atrum* (XP_003009023) for CRY-DASH; *Xenopus laevis* (NP_001089127), *Drosophila melanogaster* (NP_523653) *Monosiga brevicollis* (XP_001746666) and *Arabidopsis thaliana* (NP_849651) for CPD class II PHR; *Verticillium albo-atrum* (XP_002999933) for fungal CPD class I PHR; *Arabidopsis thaliana* (NP_567341 and NP_171935) for plant CRY and *Arabidopsis thaliana* (NP_182281) for plant PHR2. Bit score of 200 was applied as a lower threshold of sequence selection. Also, we conducted the BLASTP and TBLASTN search in different taxonomy groups of prokaryotes using the same set of queries, to clarify the existence of CRY/PHR proteins in bacteria and archaea. One PHR sequence from each bacteria (*Cronobacter sakazakii* CPD Class I PHR YP_001438714; *Gloeobacter violaceus* (6–4) PHR NP_924695; *Spirosoma linguale* CRY-DASH YP_003390944 and *Geobacter sulfurreducens* CPD Class II PHR NP_953872) and archaea (*Halorhabdus utahensis* CRY-DASH YP_003131490 and CPD Class I PHR YP_003131773; *Methanosarcina barkeri* CPD Class I PHR YP_304088) was included to represent the prokaryotic CRY/PHR groups. Finally, a total of 762 sequences were selected for the analysis (S1 Table).

RNA polymerase II subunit RPB2 was used for the comparative taxonomical analysis [26]. The *Homo sapiens* RPB2 (NP_000929) protein sequence was retrieved using the following sequence as a probe. DNA-directed RNA polymerase subunit B of 2 archaea (*Halorhabdus utahensis* and *Methanosarcina barkeri*) and 4 bacteria (*Cronobacter sakazakii*, *Geobacter sulfurreducens*, *Gloeobacter violaceus* and *Spirosoma linguale*) were used as outgroups (S2 Table). The taxonomical analysis involved smaller number of species (206) because RPB2 sequences from some species were not available in the databases.

### Sequence editing and aligning

The protein sequences were aligned by MUSCLE v. 3.8.31 [27]; the nucleotide sequences were aligned according to the protein alignment using Rev-Trans v.1.4 [28] available at http://www.cbs.dtu.dk/services/RevTrans/. The aligned sequences were trimmed manually in Bioedit v.7.0.9 [29] by removing poorly aligned terminal regions. The final alignment of the CRY/PHR and RBP2 proteins included 489 and 1172 positions, respectively. The sequences utilized in this study are listed in online supporting information S1 and S2 Tables. Also, the alignment of CRY/PHR which is utilized for further analyses was uploaded as supporting information.

### Phylogenetic reconstruction

The phylogenetic reconstruction was performed using the protein sequences. The most appropriate model of amino acid substitutions for the data set was determined according to the Akaike information criterion (AIC) and using ProtTest v.3.0 [30,31]. Based on the test, the LG model with proportion of invariable sites, gamma distribution and equilibrium frequencies (LG+I+G+F, p-inv = 0.046, α = 0.723) [32] was used for the phylogenetic analysis of the RPB2 proteins. For the CRY/PHR proteins, the best fitting model was WAG+G (α = 1.061). Two phylogenetic algorithms were utilized to infer the tree. First, the maximum likelihood (ML) phylogenetic tree was constructed using PhyML v.3.0 [33]. The approximate likelihood-ratio test (aLRT) [34] was applied to estimate a statistical support for individual nodes. Second, we used the Bayesian relaxed clock as implemented in BEAST v.2.1.3 [35]. The length of the MCMC chain was set for 10 million with trees sampled every 1000 steps. The maximum clade credibility tree was determined using TreeAnnotator v.2.1.2 from the BEAST software package.

### Analysis of selection

Gene duplication is an important mechanism for generating novel functional proteins, because the redundant homologs are free to accumulate substitutions. However, whether the new function of a gene evolves under positive selection remains controversial [36]. We analyzed positive selection using the ML approach as implemented in HyPhy software package v.2.1.2 [37]. The ratio of nonsynonymous to synonymous substitutions (dN/dS or ω) was used to measure the strength of selection on the CRY/PHR genes. The set of tests was conducted: (1) the basic model (M0), which estimates uniform ω ratio among all sites, was used to calculate a representative estimate for the whole dataset [38]; (2) the site models including M1 (nearly neutral), M2 (selection), M3 (discrete), M7 (beta distribution, ω > 1 disallowed) and M8 (beta distribution, ω > 1 allowed) [39–41]. The likelihood-ratio test (LRT) was performed between the following pairs of the models: (1) M3 vs. M0; (2) comparisons of site models including M2 vs. M1 and M7 vs. M8. Then χ^2^ tests were performed with degrees of freedom (*df*) between two compared models.

Also, we utilized the recently developed method, branch-site random effects likelihood (REL) [42] implemented in HyPhy v.2.1.2 [37], to detect episodic diversifying selection. This LRT-based approach identified all lineages in the phylogeny with a proportion of positive selected sites, without making priori assumptions (“foreground” and “background” branches) that may lead to high rates of false positive or negative selection [42]. The branch-site REL method has been successfully applied to infer positive selection in several research publications [43–45].

### Identification of conserved residues

Evolutionarily conserved residues and motifs are hypothesized to be functionally important [46]. We utilized ConSurf (http://consurf.tau.ac.il/) to identify probable functionally important residues in the CRY/PHR proteins [47]. The analysis was conducted using the Bayesian algorithm and the LG model with the parameters as specified above. The same sequence alignment used in DIVERGE v.2.0 was uploaded to the server. Degrees of conservation in the protein subfamilies visualized with Chimera v.1.6.2 [48].

### Estimating evolutionary time of gene duplications and gain-loss events

The inferred RPB2 tree was tested for the presence of molecular clock using HyPhy v.2.1.2 [37]. Based on the test results, the model with local clock was utilized for the further analysis. Six internal calibration points (CP1-CP6) were used for time estimates. CP1-CP4 indicate the origins of main groups of animals with minimum and maximum time estimates constrained by respective biostratigraphic evidence [49]: CP1 corresponds to the origin of eutherians (113–95.3 Mya); CP2 is the divergence of birds and crocodile (250.4–235 Mya); CP3 is the split of the ray-finned fishes and tetrapods (421.75–416.1 Mya) and CP4 corresponded to the divergence of flies and mosquitos (295.4–238.5 Mya). CP5 was inferred from the phylogenetic study of three genes (*rbcL*, *atpB*, 18S rDNA) in 560 angiosperms, which estimated the origin time of Angiospermae between 179 and 158 Mya [50]. CP6 is the origin of Ascomycota about 500–650 Mya [51].

The computations were conducted using the ML approach as implemented in PAML v. 4.4 [52]. The substitution model for the data was determined by jModelTest v.0.1.1 [53]. The GTR+I+G (p-inv = 0.031 and α = 0.455) model turned to fit our data best [54]. We also used the Bayesian relaxed clock as implemented in BEAST v.1.6.2 to estimate the dates of various events in evolution of the superfamily [35].

## Results

### Occurrence and phylogeny of the CRY/PHR superfamily in eukaryotes

Members of the CRY/PHR superfamily were found in genomes of species across all kingdoms; however, their occurrence greatly varied among taxa (Table 1). The most ubiquitous groups in eukaryotes are CPD class II photolyases, (6–4) photolyases, and CRY-DASH, which are present in the majority of the studied taxa. The other types of photolyases are more taxon-specific. CPD class I photolyases occur only in prokaryotes, fungi and basal eukaryotes (Ciliophora and Euglenozoa). Among fungi, Ascomycetes and Basidiomycetes possess only CPD class I but no CPD class II PHR, while unicellular *Nosema ceranae* has only CPD class II PHR. The CPD class II photolyases are ubiquitous in archaea and eukaryotes but rare in bacteria (found only in Methanomicrobia and Methanobacteria) and lost in placental mammals. Three subfamilies are specific either to plants or animals: plant cryptochromes, plant PHR2 (photolyase/blue-light receptor 2), and animal cryptochromes (Table 1).

**Table 1:** Occurrence of the CRY/PHR genes in main taxa.

Taxa	Class I CPD *Phr*	Class II CPD *Phr*	(6–4) *Phr*	*Cry-DASH*	Plant *Cry*	Plant *PHR2*	Animal *Cry*
Prokaryotes							
Archaea	+	+^a^		+^b^			
Bacteria	+	+	+^c^	+			
Alveolata							
Ciliophora	+						
Perkinsea		+					
Apicomplexa		+					
Stramenopiles							
Bacillariophyta		+	+	+			
Oomycetes			+	+			
Eustigmatophyceae			+	+			
Other basal eukaryotes							
Euglenozoa	+						
Amoebozoa		+					
Heterolobosea				+			
Rhodophyta				+			
Cryptophyta				+			
Choanoflagellates		+	+	+			
Fungi							
Microsporidia		+^d^					
Ascomycetes	+		+	+			
Basidiomycetes	+		+	+			
Viridiplantae							
Chlorophyta		+	+	+	+	+	
Bryophyta		+	+	+	+	+	
Lycopodiophyta		+	+	+	+	+	
Amborellales		+	+	+	+	+	
Liliopsida		+	+	+	+	+	
Eudicotidae		+	+	+	+	+	
Animalia							
Nematoda		+					
Cnidaria		+	+	+			+
Mollusca		+	+	+			+
Crustacea		+	+	+			+
Insecta		+	+				+
Echinodermata		+	+	+			+
Cephalochordata ^e^							+
Chondrichthyes ^e^							+
Actinopterygii		+	+	+			+
Sarcopterygii ^e^							+
Amphibia		+	+	+			+
Testudines		+	+	+			+
Lepidosauria		+	+	+			+
Archosauria		+	+				+
Aves		+	+	+			+
Metatheria		+					+
Monotremata							+
Eutheria							+

^a^: Found only in Methanomicrobia and Methanobacteria

^b^: Found only in Halobacteria

^c^: Found only in *Gloeobacter violaceus*

^d^: Found only in *Nosema ceranae*

^e^: Absent of CRY/PHR homologs may be due to insufficient annotation

**Fig 2:**
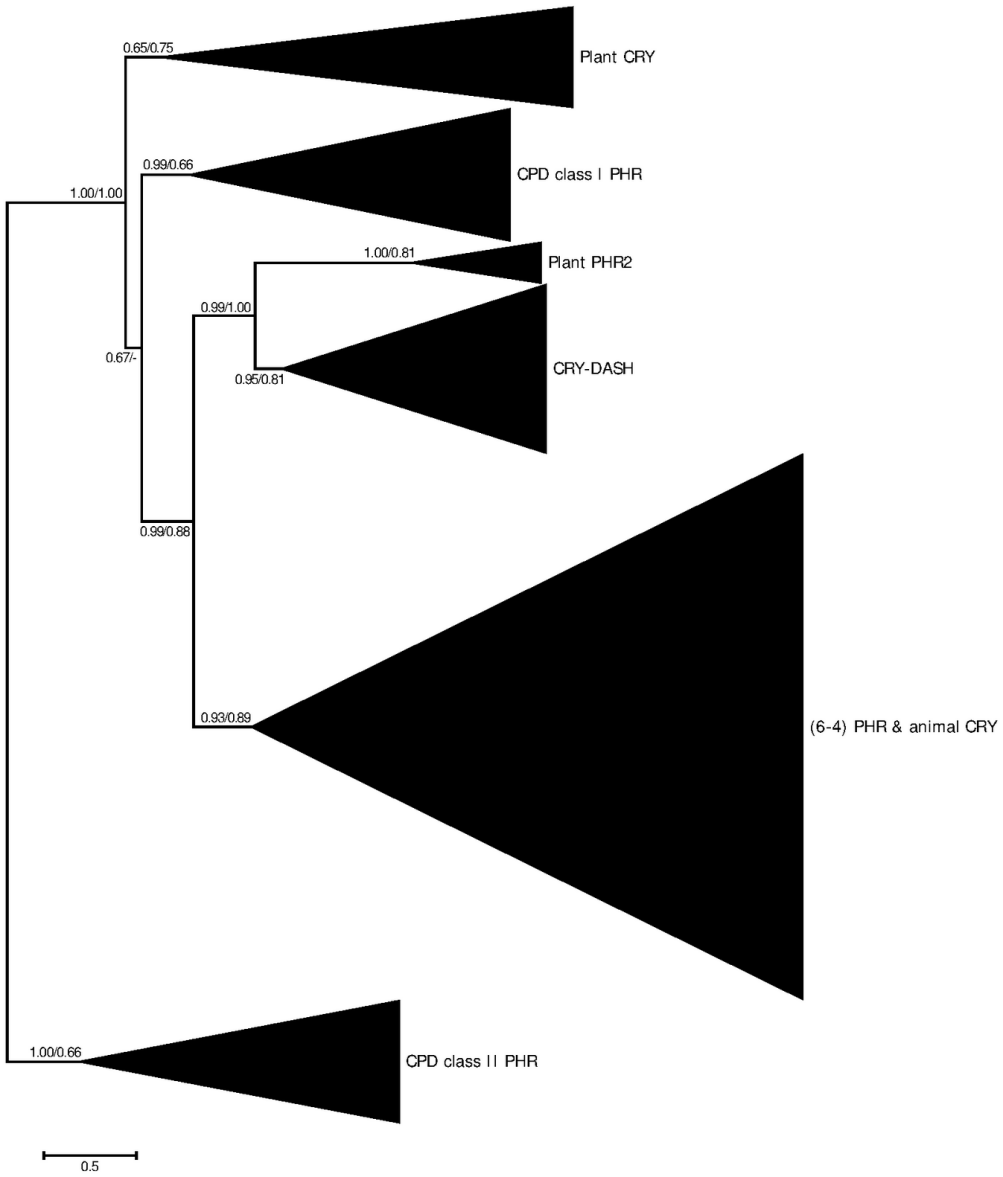
Unrooted maximum-likelihood tree of the CRY/PHR homologs. Maximum-likelihood probabilities and Bayesian posteriors of the node support below 0.5 are not shown. The values are the likelihoods and posteriors respectively. The CRY/PHR superfamily include 6 main subfamilies: animal CRY and (6–4) photolyases, CRY-DASH, plant PHR2, plant CRY and 2 classes of CPD photolyases (CPD class I and class II).

Both the ML and Bayesian trees feature 6 main clades with significant statistical support, which correspond to animal CRY and (6–4) photolyases, CRY-DASH, plant PHR2, plant CRY and 2 classes of CPD photolyases (CPD class I and class II) (Fig 2).

### Selection in the genes of the CRY/PHR superfamily

We utilized 6 models implemented in PAML to estimate the selective forces after several duplications having occurred in the CRY/PHR superfamily (Table 2). According to the results, the global ω estimated by M0 were low (0.0168), indicating that cryptochromes and photolyases have experienced strong purifying selection. Site model M2a is significantly better than M1a (2ΔlnL = 3996989.1508, *df* = 2, p < 0.01), and M8 manifests a higher likelihood as compared to M7 (2ΔlnL = 1998514.0002, *df* = 2, p < 0.01). Therefore, the alternative hypotheses (selection) cannot be rejected. However, no positively selected sites were identified by the site models.

**Table 2:** Results of the codon based positive selection tests by HyPhy.

Model	Parameters
Model 0 (one ratio)	lnL = -507700.8700
	ω = 0.0168
Model 1 (Neutral)	lnL = -397024795.0599
	ω = 0.0168 ± 0.0000
	P_0_ = 1.0000, ω_0_ = 0.0168
Model 2 (Selection)	lnL = -395026300.4845
	ω = 0.0184 ± 0.0000
	P_1_ = 1.0000, ω_1_ = 0.0184
	P_2_ = 0.6005, ω_2_ = 4.0276
LRT ^a^ (M1/M2, *df* = 2)	2ΔlnL = 3996989.1508 **
Model 3 (Discrete)	lnL = -398024989.2332ω = 0.1823 ± 0.0252
	P_1_ = 0.7792, R_1_ = 0.2038, ω_1_ = 0.4802, P_2_ = 1.0000, R_2_ = 1.4261
LRT (M0/M3, *df* = 4)	2ΔlnL < 0
Model 7 (Beta)	lnL = -398023356.0916
	ω = 0.0338 ± 0.0012
	β_P_ = 0.6156, β_Q_ = 17.5868
Model 8 (Beta & ω)	lnL = -397024099.0915
	ω = 0.0650 ± 0.0034
	β_P_ = 0.8055, β_Q_ = 11.5904, ω_1_ = 1, P = 1.0000
LRT (M7/M8, *df* = 2)	2ΔlnL = 1998514.0002 **

^a^: Likelihood ratio test was estimated by 2ΔlnL and followed by a χ^2^ test

*: p < 0.05

**: p < 0.01).

LRT = -2(lnL_0_-lnL_A_)

The branch-site REL method [42] determined about 35.67% (270/757) of the branches to evolve under episodic diversifying selection (S3 Table and S1 Fig). Some of them had sites with very large ω^+^ (>> 1.0) but the proportion of the sites under positive selection was generally less than 10% in most branches ranging between 0.021–0.082 (e.g., Node99, ω^+^ = 5230.128, weight p^+^ = 0.043). On the other hand, some lineages, e.g., plant CRY in higher plants (Node9), indicated quite a large proportion of branch length (weight p^+^ = 0.163) with positive selection (S3 Table and S1 Fig).

### Identification of conserved amino acid residues

**Fig 3:**
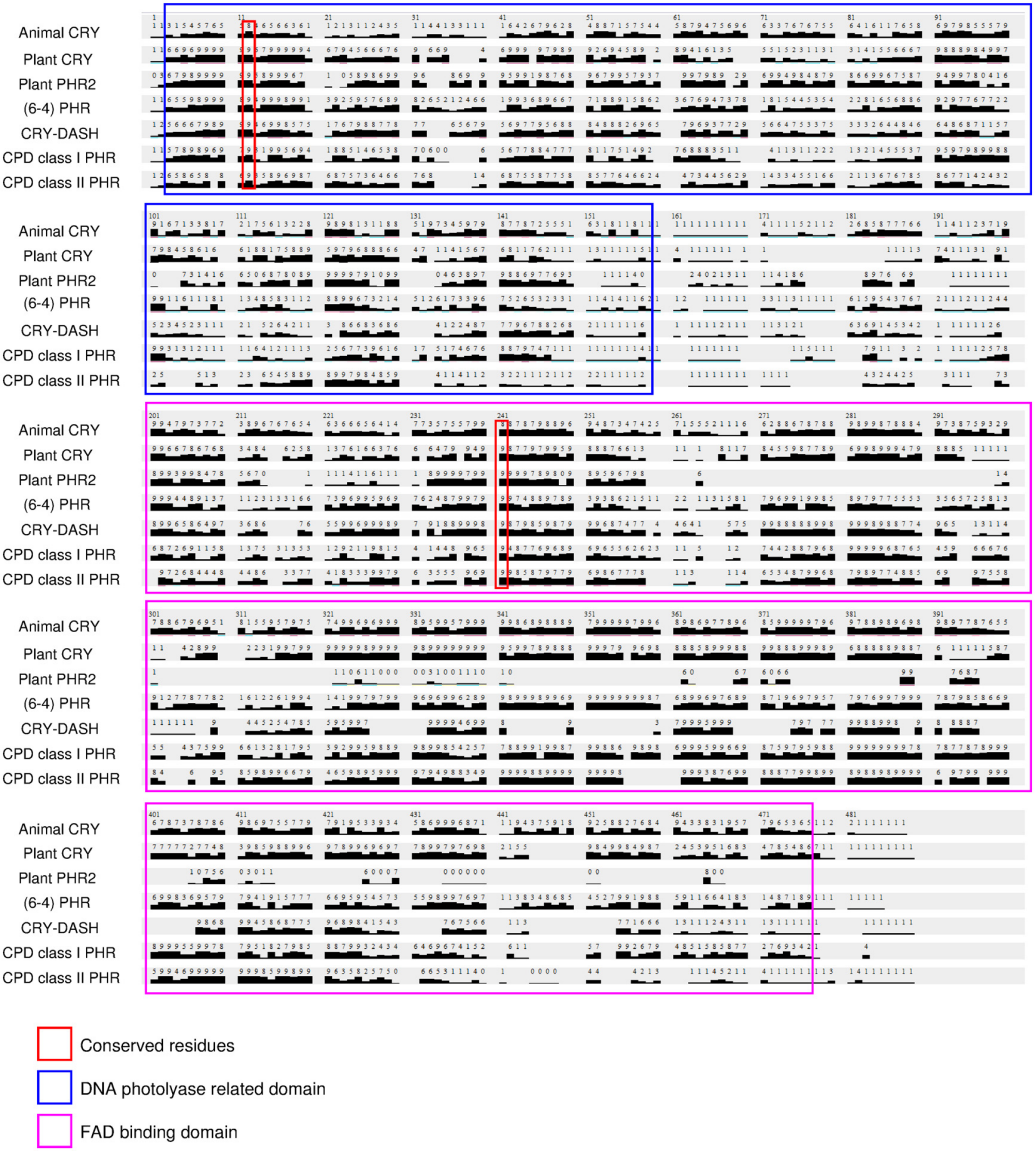
Group conserved residues identified by ConSurf. Degrees of conservation in the protein subfamilies visualized with Chimera v.1.6.2 [48]. The numbers of upper row and black bars indicate the level of conservation (0–9), 0 being the lowest.

In the CRY/PHR superfamily, clusters of conserved sites were located in DNA photolyase related domain (positions 3–159 of the alignment) and FAD binding domain (position 201–477) (Fig 3). The FAD binding domains of plant PHR2 proteins are shorter (~ 95 aa) and share fewer conserved residues than in the other subfamilies. In addition, we identified two highly conserved residues (with conservation scores ≥ 8) shared by all 7 subfamilies: the Arg locates at position 12 of the alignment and the Ser at position 241 (Fig 3).

### Time estimates of the events in CRY/PHR superfamily evolution

**Fig 4:**
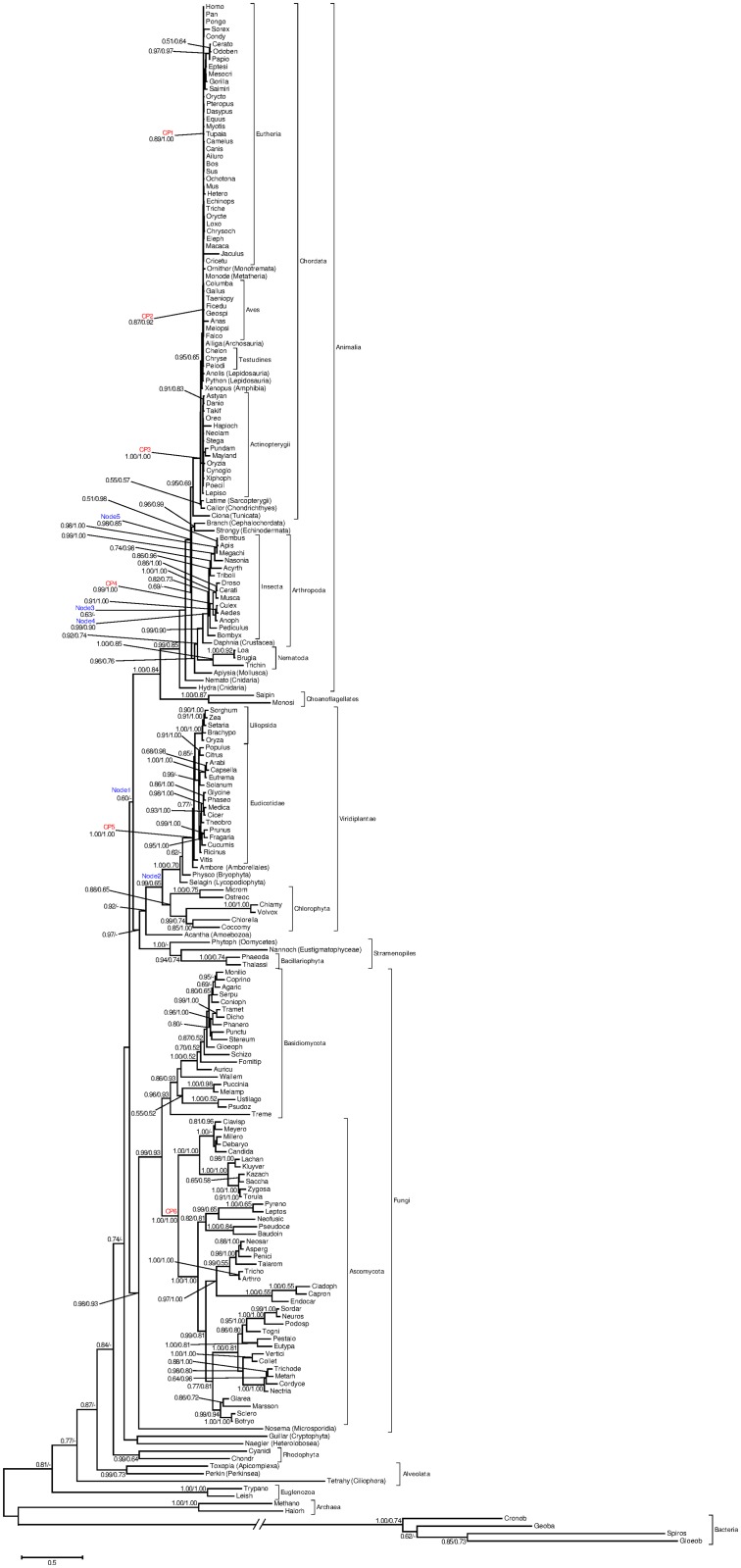
Maximum-likelihood tree with local clock of the RPB2 homologs. Maximum-likelihood probabilities and Bayesian posteriors of the node support below 0.5 are not shown. The values are the likelihoods and posteriors respectively. The internal calibration points (CP1-CP6): CP1 corresponds to the origin of eutherians (113–95.3 Mya); CP2 is the divergence of birds and crocodile (250.4–235 Mya); CP3 is the split of the ray-finned fishes and tetrapods (421.75–416.1 Mya) and CP4 corresponded to the divergence of flies and mosquitos (295.4–238.5 Mya). CP5 estimated the origin time of Angiospermae (179–158 Mya) [48,87]. CP6 is the origin of Ascomycota about 500–650 Mya [49]. In addition, time of five evolutionary events were estimated (Node11-5). Time estimates was showed in Table 3.

Time estimates of the key events in evolution of the CRY/PHR superfamily are given in Table 3 and Fig 4. Generally, the ML analysis yielded the smaller values as compared to the Bayesian analysis. On the other hand, the ML estimates fall within the 95% HPD range of the Bayesian estimates. The aggregated results from both analyses suggest that several major gene duplication and losses in the superfamily occurred between 700 and 400 Mya (Table 3), which correspond to four geological periods: Devonian, Silurian, Ordovician, and Cambrian. The origin of animal CRY is dated back to the Neoproterozoic Era (~1000–541 Mya). The plant CRY is evolutionary younger and evolved in Paleozoic Era (~541–252) (Table 3).

**Table 3:** Maximum-likelihood and Bayesian time estimates for the nodes (Mya) (Fig 4).

Node	Maximum Likelihood	Bayesian ^b^	Evolutionary Events
1	812.23 ± 142.23	961.33 (755.41–1359.82)	Loss of CPD class I *Phr* in plants and animals
2	528.21 ± 168.73	422.30 (285.08–679.26)	Origin of plant *Cry*
3	606.62 ± 122.43	698.45 (500.62–769.12)	Origin of animal *Cry*
4	-^a^	320.27 (269.12–408.18)	Loss of *Cry-DASH* in insects
5	544.06 ± 120.83	598.85 (482.22–638.85)	Origin of *Cry4*
CP1	113–95.3		Loss of photolyases, *Cry-DASH* and *Cry4* in placental mammals [47]
CP2	86.5–66		Loss of *Cry-DASH* and (6–4) *Phr* in birds [47]
CP3	421.75–416.1		Duplication of vertebrate *Cry* [47]
CP4	295.4–238.5		Loss of insect *Cry2* in fly [47]
CP5	179–158		Duplication of plant *Cry* [48,87]
CP6	500–650		Origin of Angiospermae [49]

^a^: Cannot be calculated due to the lack of the DNA sequence of the *Daphnia pulex* RPB2 (EFX81055)

^b^: Posterior mean (95% HPD)

## Discussion

### Origin and evolutionarily ancient diversification of photolyases

The phylogenetic tree of the CRY/PHR proteins inferred in the present study was similar to those from the previous studies [5,11]. The protein subfamilies form several main clades, including animal CRY & (6–4) photolyases, CRY-DASH, plant CRY and 2 classes of CPD photolyases (CPD class I and class II). Also, we identified a new group of photolyases, PHR2, which is specific to plants. The PHR2 homologs formed a clade phylogenetically related to CRY-DASH.

The pioneering phylogenetic study of cryptochrome/photolyase superfamily [23] involved limited number of sequences. In that study, the CPD class I PHR photolyases were classified into two types according to their cofactors, MTHF and 8-HDF [23]. The split of these two types was thought to occur in the early stage of the evolution of the CRY/PHR superfamily (before the divergence of prokaryotic and eukaryotic organisms) [23].

The occurrence of CRY/PHR proteins are taxon-specific (Table 1). The distribution of the CRY/PHR proteins in protists reflects paraphyly and evolutionary diversification of this group of eukaryotes. The evolutionary oldest protists, *Trypanosoma brucei*, *Tetrahymena thermophile* and *Leishmania major* have only CPD class I. The evolutionary younger protists lack CPD class I and may possess members of the other three types of photolyases (CPD class II, (6–4) PHR and CRY-DASH).

Another interesting fact is that mammals possess no CPD class II photolyases, except for marsupials (*Monodelphis domestica* and *Sarcophilus harrisii*). Recent research demonstrated that the CPD photolyase in a marsupial *Potorous tridactylus* was able to act as a cryptochrome, suggesting that ancestral CRY/PHR proteins were likely manifested both DNA repair and circadian clock function [55].

The PHR2 (photolyase/blue receptor 2) subfamily previously reported from green algae *Chlamydomonas reinhardtii* [56] is common in higher plants (S1 and S2 Figs). However, they are absent from all other algae (S1 Table). The PHR2 protein from *Chlamydomonas* manifests CPD photolyase repairing activity for both chloroplast and nuclear DNA [56]. However, PHR2 in *Arabidopsis thaliana* shows no chloroplast DNA repair activity [57]. At the same time, the CPD class II PHR of higher plants exhibits the CPD repair function in DNA of all cellular genomes (nuclear, chloroplasts and mitochondrial) [58]. The property of photolyases to target different organelles is thought to be associated with some functional motif, but the exact mechanism of organelle-targeting is yet to be determined [58]. Among eukaryotes, plants have the largest set of various types of photolyases (Table 1). Some of them have similar functions, as in the above example. Such functional redundancy may be an evolutionary adaptation to the relatively high, as compared to the other eukaryotes, exposure to sunlight and, respectively, harmful UV radiation. This higher exposure prompts for more efficient DNA repairing mechanism.

Although the physiological function of PHR2 is similar to that of the CPD class I and CPD class II proteins, their sequence homology is weak. Our results suggest that PHR2 likely evolved from an ancient CRY-DASH gene (Fig 2). This split was followed by significant functional divergence: CRY-DASH encodes a single-strand DNA (ssDNA) CPD photolyase, whereas plant PHR2 repairs CPD dimers of double-strand DNA (dsDNA) [1]. The ssDNA binding property of CRY-DASH is closely related to its structure, the CPD-binding cavity of the *Arabidopsis* CRY-DASH is unable to stably bind CPD from the dsDNA since the binding is less energetic [59].

Based on their roles in the circadian clock, two groups of functionally different animal CRY proteins were identified [60]. A *Drosophila*-like type 1 CRY is a UV-A/blue light receptor in the circadian oscillator, while a vertebrate-like type 2 CRY is thought to be a negative regulator of the clock’s transcriptional feedback loop [61]. Whether the type 2 cryptochromes also have photoreceptor function is still debated [11]. There are several transcription factors involved in animal circadian feedback loop, including Period (PER), Timeless (TIM), brain and muscle Arnt-like protein-1 (BMAL1), CYC (Cycle) and circadian locomotor output cycles kaput (CLOCK or CLK) [62–66]. The molecular mechanisms of the fly and mammalian circadian clocks are different. The fly CRY binds to TIM that results in degradation of the latter and subsequent inhibition of the activity of a PER/TIM dimer. Without CRY, the PER/TIM dimer is able to enter nucleus and repress the transcription of other clock genes. In mammals, three period (PER 1, 2 and 3) and two cryptochrome (CRY 1 and 2) proteins form a cytoplasmic heterodimer, which enters the nucleus and then inhibits transcription of CLK and BMAL1 [67]. In plants, circadian clocks are entrained by red light receptor phytochromes (PHY) as well as blue light receptor cryptochromes, but the signal transduction pathways were not comprehensively studied [68].

The obtained phylogenetic tree (S1 and S2 Figs) indicates that there are two insect *Cry* paralogs, *Cry1* and *Cry2*. Unlike the evolutionary older CRY1, CRY2 is not photosensitive and has a transcriptional repressive function similarly to vertebrate CRY [69]. Among the studied insect species, only few possess both paralogs, the others lack either one. Interestingly, the majority of insects lost photosensitive CRY1 and has only CRY2. It is still unknown how insects lacking CRY1 sense light. It may be possible that those species have developed some compensating mechanism for photosensitivity.

The duplication of the insect CRY ancestor probably occurred well before the origin of insects, maybe even prior to the split of deuterostomes and protostomes, because homologous cryptochromes were found in *Nematostella vectensis* (Nemato1, Nemato1b, Nemato1c and Nemato1d), *Strongylocentrotus purpuratus* (Strongy1 and Strongy2) and *Daphnia pulex* (DaphniaM and DaphniaD, S1 Fig). The duplications of cytochromes also occurred in vertebrates and plants, but there is no evidence in the available literature that the paralogs of mammalian and plant CRYs have experienced functional divergence.

The genome of zebra fish *Danio rerio* possesses the largest known number of the cryptochrome genes, 8. Four of them (NP_001070765, NP_571865, NP_571866 and NP_571867; annotated as *Cry1a*, *1b*, *2a* and *2b*, respectively) are very similar and appear in the same clade with vertebrate *Cry1*; one (BAA96850) annotated as *Cry3* falls in the clade with vertebrate *Cry2*, one (NP_571862) is *Cry4*, one (NP_991249) is *Cry-DASH* and one (XP_009291670) is plant-like *Cry* [70]. Recently, a plant-like *Cry* was identified in *D*. *rerio* (XP_009291670), which may act as a circadian photoreceptor [70]. The maximum number of cryptochrome gene copies in other animal taxa (including birds, reptiles and amphibians) is usually 2 or 3. The extra *Cry* homologs in zebra fish were likely generated by ancient polyploidy events [71]. In the evolution history of vertebrates, their genomes were duplicated twice (occurred in the early evolution of deuterostomes), and a third genome duplication, which is named the fish-specific genome duplication (FSGD), occurred in the basal group of ray-finned fishes (Actinopterygii) (~ 350 Mya) [72]. The *Cry4* genes are apparently evolutionarily oldest among animal cryptochromes. A primitive Cephalochordate *Branchiostoma floridae* possesses homologs of *Cry4* (XP_002609503 and XP_002595074), thus suggesting that *Cry4* might emerge in Chordata, and then be lost in mammals. However, CRY4 was reported to exhibit neither (6–4) PHR nor circadian functions [73,74].

Cryptochromes of *Drosophila* (insect CRY1) and plants exhibit similar physiological functions, both of them play roles in light sensing and entrainment of circadian oscillator, but are not essential for the self-regulation of the clock [75,76]. However, the evolutionary history of plant and animal cryptochromes is quite different: they evolved from the different ancestral photolyase genes soon after the plant—animal divergence [77]. Animal cryptochromes originated from (6–4) photolyases, while plant cryptochromes are evolved from CPD photolyases (Fig 2). Given that cryptochromes (except for CRY-DASH) are absent in fungi and prokaryotes, this may suggest that the plant and animal cryptochromes might appear soon after the origin of these taxa [77]. The origin of animal *Cry* was accompanied by coevolution of other circadian components including PER, BMAL1, CYC and CLK [77].

Photolyases, including CPD PHR, (6–4) PHR and single-strand photolyase CRY-DASH, were lost in higher animals, including placental mammals (Fig 2). The loss of PHR genes in higher animals was thought to occur due to weak natural selection [5]. In the absence of photolyases, placental mammals rely on more complex and less efficient nucleotide excision system consisting of DNA glycosylases, nucleases and DNA polymerases to eliminate pyrimidine dimers [5,78]. It was hypothesized that the loss of photolyases in higher eukaryotes was associated with the reduced UV stress [5]. Strong UV radiation was diminished by accumulation of oxygen since Proterozoic (~ 2500–540 Mya) [79].

Although eukaryotes and some prokaryotes (cyanobacteria) display circadian rhythms, the input signals to the clock are not always controlled by cryptochromes. In plants, circadian rhythms are activated through the red light receptor phytochrome (PHY) and blue light receptor cryptochrome [80]. The generic feedback loop of circadian rhythm in fungi is different: blue light is absorbed by the flavoprotein white collar-1 (WC-1) [81]. Cyanobacteria are the simplest organisms to exhibit circadian rhythmicity [82]; an environmental signal is transduced to the endogenous clock by the circadian input kinase A (CikA) [83], and the whole circadian system is controlled by the *kaiABC* gene cluster [84]. There had been no evidence for common ancestor of eukaryotic and prokaryotic circadian genes [85], until it was found that the cryptochromes have a common ancestor with the prokaryotic photolyase [17]. However, it is still unclear what circadian function is performed by CRY-DASH in prokaryotes. Another open question is how the distinctive circadian mechanisms emerged in different groups of eukaryotes. This prompts for further extensive studies of circadian genes in prokaryotes and eukaryotes to solve the above problems.

### Episodic positive selection and conservation of photolyase and cryptochrome

The results of the selection analysis are in an agreement with the previously reported data, indicating that CRY/PHR genes have evolved primarily under strong purifying selection (Table 2) [5,86]. On the other hand, positive selection likely occurred during some periods of cryptochromes’ evolution: the members of the superfamily experienced multiple duplications and neofunctionalizations, which are usually accompanied by strong episodic positive selection [87]. The site-specific models are conservative in estimating positive selection, especially for protein superfamilies with long evolutionary histories [88]. Therefore, we utilized the branch-site model to detect episodic diversifying selection.

Based on the results of REL analysis, episodic diversifying selection was apparently quite common in the evolution of the PHR/CRY superfamily (S3 Table and S1 Fig). In most cases, episodic diversifying selection operated on speciation events, e.g., plant CRYs of Angiospermae. Angiosperms are an evolutionary young (~179–158 Mya) and species-rich group, which experienced fast diversification and dominated almost all environments on Earth [89]. The speciation processes were likely followed by episodic diversifying selection in circadian genes, which might help these species to adapt to various ecological niches. Similar scenario was reported for molecular evolution of other eukaryotic circadian genes, particularly plant phytochromes (PHY) [90]. This might imply coevolution of cryptochromes and phytochromes. Indeed, phytochromes directly or indirectly interact with CRY and perform similar circadian functions (red/far-red light receptors), thus PHY and CRY might evolve under similar selection pressure [91]. On the other hand, strong positive selection played a role in functional divergence of PHR/CRY proteins, e.g., Node 320 (divergence of CRY-DASH and plant PHR2) (S1 Fig).

The overall sequence similarity between the two homologous domains, DNA photolyase related domain (positions 3–159 of the alignment) and FAD binding domain (position 201–477), is generally high (Fig 3). The observed high conservation of PHR/CRY sequences corresponds to the fact that PHR and CRY proteins maintain a conserved 3D structure [11]. On the other hand, only two conserved residues shared by all subfamilies (Fig 3). One of them, Arg, is located at position 12 of the alignment, which was reported within the binding site of Cl^-^ in *Arabidopsis* CRY-DASH (R51 of PDB: 2VTB) [92]. As to S241, no functional or structural importance of this residue was reported previously. S241 is adjacent to α helices; we hypothesize that this residue is essential for maintaining the secondary structure of α helix and, respectively, for FAD binding (e.g., S260 and α14 of *Arabidopsis* CRY1, PDB: 1U3D [93]). In addition, the FAD binding domains of plant PHR2 are shorter and less conserved as compare to the other subfamilies. It is yet to be studied how plant PHR2 maintains its function with such an "incomplete" domain.

### The key events in CRY/PHR evolution and major changes in the global light regime

The phylogenetic analysis along with the biostratigraphy made it possible to estimate the approximate time of key events in evolution of the CRY/PHR superfamily. According to the time estimates based on the ML and Bayesian approaches, plant, insect and vertebrate cryptochromes originated in Neoproterozoic Era (~1000–541 Mya). Studies of fossil record and geological patterns suggest that, during that period, the day length steadily increased from 18 h at 900 Mya [94] to 21 h at 600 Mya [95] and to approximately 22 hours at 650–600 Mya [96]. An ancestor of the vertebrate *Cry1* and *Cry2* duplicated in Silurian-Devonian Period (~443.4–358.9 Mya) (Table 3 and Fig 4). At that time, the climate of Earth became stable and warm, the concentration of oxygen increased and the level of the harmful UV radiation lowered [97]. New groups of living organisms evolved and spread in this era, such as lobe-finned fish and amphibians [98]. As one of the main adaptation mechanisms, the endogenous circadian system increases Darwinian fitness through synchronizing the metabolic and other biological rhythms of an organism with environmental light/dark cycle [99]. Therefore, the circadian system in eukaryotes might have experienced certain evolutionary changes to adjust to the increase of day length. These changes might include, among the others, functionally important substitutions in the existing circadian genes, the origin of clock genes *de novo* or co-option of non-circadian genes to perform circadian function. In these terms, the general direction of the circadian system evolution in eukaryotes is similar to that in prokaryotes (cyanobacteria) [100]. Furthermore, the time estimates for the major events in evolution of cryptochromes and the cyanobacterial circadian system [101] correspond to each other and to the major environmental changes in the global light regime. For example, the origin of animal cryptochromes (elements of the circadian input pathway) occurred about the same time (~700–600 Mya) when the *bona fide* circadian system of some cyanobacteria lost *kaiA*, also an important element of the circadian input [101], In turn, this period corresponds to the suggested upper time limit of the last of the three periods proposed to describe the role of UV radiation in the evolution of cyanobacteria [102].

Circadian rhythmicity and photo-activated DNA repair were suggested to have a common evolutionary origin [103]. Escape from sunlight represented a major selective force for development of circadian rhythms [104]. Geological studies indicated that in Precambrian times (~3800–544 Mya) atmosphere contained little oxygen and primitive organisms were exposed to high ultraviolet radiation during the daytime [105]. There are 2 main strategies for organisms to avoid the harmful effects of UV radiation [6]. The first one is repairing the UV-induced DNA damage which is the physiological function of photolyase. The other one is to avoid being irradiated, such as migrate to deeper water. These movements were observed by the diel vertical migrations of zooplankton, which initiated and controlled by light [106]. Such migrations also occur in other marine and freshwater organisms such as water flea *Daphnia magna* [107], and sensitivity was related closely to the UV photoreceptors in its compound eye [107]. These diel vertical migrations may help to understand the coevolution of photoreception and circadian rhythms, and the coevolution of their respective controlling genes [6].

## Supporting Information

S1 Fig Unrooted maximum-likelihood tree of the CRY/PHR homologs. Maximum-likelihood probabilities of the node support below 0.5 are not shown. The values are the likelihoods and bootstraps respectively. Internal branch marks in blue font (e.g. Node14) correspond to branches evolved under episodic diversifying selection; marks in violet font (e.g. Node6) represent positively selected branches (mean ω > 1) (S3 Table). The bar below the tree indicates 0.5 substitutions per site.
(JPG)

S2 Fig Unrooted Bayesian tree of the CRY/PHR homologs. The node labels are the posterior values. The branches width and color correspond to the substitution rate. The bar below the tree indicates 0.5 substitutions per site. Relative evolutionary rates are illustrated by color and width of the branches. Thinner-thicker and lighter-darker indicate lower-higher rates, respectively.
(JPG)

S1 Table List of the CRY and PHR sequences used in the study. (DOCX)

S2 Table List of the RPB2 sequences used in the study. (DOCX)

S3 Table Results of episodic diversifying selection test by REL. Mean ω was obtained using local MG94 model (no site-to-site variation). Values of ω^+^ and weight ω^+^ reflect the strength of selection and the proportion of the total branch length of positive selection. Estimates of the uncorrected *p*-value were generated by the mixture of distributions, corrected *p* was the probability obtained after Holm’s correction for multiple testing. Branches with mean ω > 1 are shaded. Infinite mean ω for branches may be resulted by lack of synonymous substitution. As a result, they were not considered to be positively selected branches.
(DOCX)
