# Supplementary material for: Evolutionary History of the Photolyase/Cryptochrome Superfamily in Eukaryotes
Source: PLoS One. 2015 Sep 9;10(9):e0135940. doi: 10.1371/journal.pone.0135940 (PMC4564169; doi:10.1371/journal.pone.0135940)
Supplement: S1 Table — (DOCX) [file pone.0135940.s003.docx]

#### S1 Table – List of CRY and PHR sequences used in the study.

| Groups | | Species and taxa | Length (bp) | GenBank nucleotide sequence accession number | Protein accession number | Designation of sequence in figure | Legend |
| --- | --- | --- | --- | --- | --- | --- | --- |
| Chordata (Animalia) | Eutheria | *Ailuropoda melanoleuca* | 1764 | XM_002927658 | XP_002927704 | Ailuro1 | Vertebrate CRY1 |
|  |  |  | 1746 | XM_002922310 | XP_002922356 | Ailuro2 | Vertebrate CRY2 |
|  |  | *Balaenoptera acutorostrata* | 1764 | XM_007165725 | XP_007165787 | Balaeno1 | Vertebrate CRY1 |
|  |  |  | 1386 | XM_007181122 | XP_007181184 | Balaeno2 | Vertebrate CRY2 |
|  |  | *Bubalus bubalis* | 1764 | XM_006069566 | XP_006069628 | Bubalus1 | Vertebrate CRY1 |
|  |  |  | 1863 | XM_006042441 | XP_006042503 | Bubalus2 | Vertebrate CRY2 |
|  |  | *Bos taurus* | 1764 | NM_001105415 | NP_001098885 | Bos1 | Vertebrate CRY1 |
|  |  |  | 1758 | NM_001289786 | NP_001276715 | Bos2 | Vertebrate CRY2 |
|  |  | *Callithrix jacchus* | 1761 | XM_002752946 | XP_002752992 | Callith1 | Vertebrate CRY1 |
|  |  |  | 1782 | XM_002755209 | XP_002755255 | Callith2 | Vertebrate CRY2 |
|  |  | *Camelus ferus* | 1764 | XM_006175444 | XP_006175506 | Camelus1 | Vertebrate CRY1 |
|  |  |  | 1386 | XM_006195723 | XP_006195785 | Camelus2 | Vertebrate CRY2 |
|  |  | *Canis lupus familiaris* | 1764 | XM_857660 | XP_862753 | Canis1 | Vertebrate CRY1 |
|  |  |  | 1440 | XM_005631304 | XP_005631361 | Canis2 | Vertebrate CRY2 |
|  |  | *Capra hircus* | 1287 | XM_005680582 | XP_005680639 | Capra1 | Vertebrate CRY1 |
|  |  |  | 1617 | XM_005709421 | XP_005709478 | Capra2 | Vertebrate CRY2 |
|  |  | *Cavia porcellus* | 1830 | XM_003462322 | XP_003462370 | Cavia1 | Vertebrate CRY1 |
|  |  |  | 1782 | XM_003465141 | XP_003465189 | Cavia2 | Vertebrate CRY2 |
|  |  | *Ceratotherium simum simum* | 1764 | XM_004418068 | XP_004418125 | Cerato1 | Vertebrate CRY1 |
|  |  |  | 1809 | XM_004436985 | XP_004437042 | Cerato2 | Vertebrate CRY2 |
|  |  | *Chinchilla lanigera* | 1764 | XM_005374636 | XP_005374693 | Chinchi1 | Vertebrate CRY1 |
|  |  |  | 1782 | XM_005384066 | XP_005384123 | Chinchi2 | Vertebrate CRY2 |
|  |  | *Chlorocebus sabaeus* | 1761 | XM_008004554 | XP_008002745 | Chloroce1 | Vertebrate CRY1 |
|  |  |  | 1782 | XM_008000546 | XP_007998737 | Chloroce2 | Vertebrate CRY2 |
|  |  | *Chrysochloris asiatica* | 1764 | XM_006865247 | XP_006865309 | Chrysoch1 | Vertebrate CRY1 |
|  |  |  | 1782 | XM_006865042 | XP_006865104 | Chrysoch2 | Vertebrate CRY2 |
|  |  | *Condylura cristata* | 1758 | XM_004675718 | XP_004675775 | Condy1 | Vertebrate CRY1 |
|  |  |  | 1783 | XM_004682881 | XP_004682938 | Condy2 | Vertebrate CRY2 |
|  |  | *Cricetulus griseus* | 1764 | XM_007615932 | XP_007614122 | Cricetu1 | Vertebrate CRY1 |
|  |  |  | 1734 | XM_007634409 | XP_007632599 | Cricetu2 | Vertebrate CRY2 |
|  |  | *Dasypus novemcinctus* | 1764 | XM_004473303 | XP_004473360 | Dasypus1 | Vertebrate CRY1 |
|  |  |  | 981 | XM_004457886 | XP_004457943 | Dasypus2 | Vertebrate CRY2 |
|  |  |  | 828 | XM_004466563 | XP_004466620 | Dasypus2b | Vertebrate CRY2 |
|  |  | *Echinops telfairi* | 1764 | XM_004700327 | XP_004700384 | Echinops1 | Vertebrate CRY2 |
|  |  |  | 1792 | XM_004708427 | XP_004708484 | Echinops2 | Vertebrate CRY2 |
|  |  | *Elephantulus edwardii* | 1755 | XM_006897551 | XP_006897613 | Eleph1 | Vertebrate CRY1 |
|  |  |  | 1782 | XM_006896741 | XP_006896803 | Eleph2 | Vertebrate CRY2 |
|  |  | *Eptesicus fuscus* | 1764 | XM_008144715 | XP_008142937 | Eptesi1 | Vertebrate CRY2 |
|  |  |  | 1788 | XM_008146774 | XP_008144996 | Eptesi2 | Vertebrate CRY2 |
|  |  | *Equus caballus* | 1764 | XM_001499213 | XP_001499263 | Equus1 | Vertebrate CRY1 |
|  |  |  | 1587 | XM_005598119 | XP_005598176 | Equus2 | Vertebrate CRY2 |
|  |  | *Erinaceus europaeus* | 1584 | XM_007515785 | XP_007515847 | Erina1 | Vertebrate CRY1 |
|  |  |  | 1788 | XM_007519095 | XP_007519157 | Erina2 | Vertebrate CRY2 |
|  |  | *Felis catus* | 1764 | XM_003989209 | XP_003989258 | Felis1 | Vertebrate CRY1 |
|  |  |  | 1746 | XM_003993282 | XP_003993331 | Felis2 | Vertebrate CRY2 |
|  |  | *Gorilla gorilla gorilla* | 1752 | XM_004053843 | XP_004053891 | Gorilla1 | Vertebrate CRY1 |
|  |  |  | 1845 | XM_004050996 | XP_004051044 | Gorilla2 | Vertebrate CRY2 |
|  |  | *Heterocephalus glaber* | 1764 | XM_004905851 | XP_004905908 | Hetero1 | Vertebrate CRY1 |
|  |  |  | 1782 | XM_004851964 | XP_004852021 | Hetero2a | Vertebrate CRY2 |
|  |  |  | 1782 | XM_004879612 | XP_004879669 | Hetero2b | Vertebrate CRY2 |
|  |  | *Homo sapiens* | 1761 | NM_004075 | NP_004066 | Homo1 | Vertebrate CRY1 |
|  |  |  | 1845 | NM_021117 | NP_066940 | Homo2 | Vertebrate CRY2 |
|  |  | *Jaculus jaculus* | 1764 | XM_004650246 | XP_004650303 | Jaculus1 | Vertebrate CRY1 |
|  |  |  | 1758 | XM_004657186 | XP_004657243 | Jaculus2 | Vertebrate CRY2 |
|  |  | *Leptonychotes weddellii* | 1764 | XM_006731050 | XP_006731113 | Leptony1 | Vertebrate CRY1 |
|  |  |  | 1782 | XM_005330030 | XP_005330087 | Leptony2 | Vertebrate CRY2 |
|  |  | *Loxodonta africana* | 1752 | XM_003405313 | XP_003405361 | Loxo1 | Vertebrate CRY1 |
|  |  |  | 1782 | XM_003412109 | XP_003412157 | Loxo2 | Vertebrate CRY2 |
|  |  | *Macaca mulatta* | 1761 | NM_001194159 | NP_001181088 | Macaca1 | Vertebrate CRY1 |
|  |  |  | 1782 | XM_001113162 | XP_001113162 | Macaca2 | Vertebrate CRY2 |
|  |  | *Mesocricetus auratus* | 1731 | XM_005083815 | XP_005083872 | Mesocri1 | Vertebrate CRY1 |
|  |  |  | 1779 | XM_005064896 | XP_005064953 | Mesocri2 | Vertebrate CRY2 |
|  |  | *Microtus ochrogaster* | 1743 | XM_005358253 | XP_005358310 | Microtus1 | Vertebrate CRY1 |
|  |  |  | 1779 | XM_005364131 | XP_005364188 | Microtus2 | Vertebrate CRY2 |
|  |  | *Mus musculus* | 1821 | NM_007771 | NP_031797 | Mus1 | Vertebrate CRY1 |
|  |  |  | 1779 | NM_009963 | NP_034093 | Mus2 | Vertebrate CRY2 |
|  |  | *Mustela putorius* | 1764 | XM_004743077 | XP_004743134 | Mustela1 | Vertebrate CRY1 |
|  |  |  | 1788 | XM_004790429 | XP_004790486 | Mustela2 | Vertebrate CRY2 |
|  |  |  | 1653 | XM_004763173 | XP_004763230 | Mustela2b | Vertebrate CRY2 |
|  |  | *Myotis davidii* | 1764 | XM_006769330 | XP_006769393 | Myotis1 | Vertebrate CRY1 |
|  |  |  | 1386 | XM_006772253 | XP_006772316 | Myotis2 | Vertebrate CRY2 |
|  |  | *Nomascus leucogenys* | 1761 | XM_003269977 | XP_003270025 | Nomascus1 | Vertebrate CRY1 |
|  |  |  | 1632 | XM_003254650 | XP_003254698 | Nomascus2 | Vertebrate CRY2 |
|  |  | *Ochotona princeps* | 1764 | XM_004589573 | XP_004589630 | Ochotona1 | Vertebrate CRY1 |
|  |  |  | 1770 | XM_004585377 | XP_004585434 | Ochotona2 | Vertebrate CRY2 |
|  |  | *Octodon degus* | 1764 | XM_004623931 | XP_004623988 | Octodon1 | Vertebrate CRY1 |
|  |  |  | 1782 | XM_004627013 | XP_004627070 | Octodon2 | Vertebrate CRY2 |
|  |  | *Odobenus rosmarus* | 1605 | XM_004412550 | XP_004412607 | Odoben1 | Vertebrate CRY1 |
|  |  |  | 1782 | XM_004399575 | XP_004399632 | Odoben2 | Vertebrate CRY2 |
|  |  | *Orcinus orca* | 1764 | XM_004269411 | XP_005979412 | Orcinus1 | Vertebrate CRY1 |
|  |  |  | 1782 | XM_004264040 | XP_004264088 | Orcinus2 | Vertebrate CRY2 |
|  |  | *Orycteropus afer* | 1764 | XM_007948230 | XP_007946421 | Orycte1 | Vertebrate CRY1 |
|  |  |  | 1770 | XM_004585377 | XP_004585434 | Orycte2 | Vertebrate CRY2 |
|  |  | *Oryctolagus cuniculus* | 1764 | XM_002711421 | XP_002711467 | Orycto1 | Vertebrate CRY1 |
|  |  |  | 1788 | XM_008270202 | XP_008268424 | Orycto2 | Vertebrate CRY2 |
|  |  | *Otolemur garnettii* | 1764 | XM_003783274 | XP_003783322 | Otolemur1 | Vertebrate CRY1 |
|  |  |  | 1839 | XM_003781259 | XP_003781307 | Otolemur2 | Vertebrate CRY2 |
|  |  | *Ovis aries* | 1764 | NM_001129735 | NP_001123207 | Ovis1 | Vertebrate CRY1 |
|  |  |  | 1791 | NM_001129736 | NP_001123208 | Ovis2 | Vertebrate CRY2 |
|  |  | *Pan troglodytes* | 1761 | XM_509339 | XP_509339 | Pan1 | Vertebrate CRY1 |
|  |  |  | 1845 | XM_001160595 | XP_001160595 | Pan2 | Vertebrate CRY2 |
|  |  | *Panthera tigris altaica* | 1764 | XM_007096456 | XP_007096518 | Panth1 | Vertebrate CRY1 |
|  |  |  | 1644 | XM_007082184 | XP_007082246 | Panth2 | Vertebrate CRY2 |
|  |  | *Pantholops hodgsonii* | 1764 | XM_005958529 | XP_005958591 | Pantholo1 | Vertebrate CRY1 |
|  |  |  | 1386 | XM_005979350 | XP_005979412 | Pantholo2 | Vertebrate CRY2 |
|  |  | *Papio anubis* | 1761 | XM_003907096 | XP_003907145 | Papio1 | Vertebrate CRY1 |
|  |  |  | 1782 | XM_003909984 | XP_003910033 | Papio2 | Vertebrate CRY2 |
|  |  | *Peromyscus maniculatus* | 1764 | XM_006986962 | XP_006987024 | Peromy1 | Vertebrate CRY1 |
|  |  |  | 1779 | XM_006995155 | XP_006995217 | Peromy2 | Vertebrate CRY2 |
|  |  | *Physeter catodon* | 1588 | XM_007101348 | XP_007101410 | Physeter1 | Vertebrate CRY1 |
|  |  |  | 1782 | XM_007124976 | XP_007125038 | Physeter2 | Vertebrate CRY2 |
|  |  | *Pongo abelii* | 1761 | XM_002823690 | XP_002823736 | Pongo1 | Vertebrate CRY1 |
|  |  |  | 1845 | XM_002821831 | XP_002821877 | Pongo2 | Vertebrate CRY2 |
|  |  | *Pteropus alecto* | 1776 | XM_006914512 | XP_006914574 | Pteropus1 | Vertebrate CRY1 |
|  |  |  | 1782 | XM_006908153 | XP_006908215 | Pteropus2 | Vertebrate CRY2 |
|  |  | *Rattus norvegicus* | 1767 | NM_198750 | NP_942045 | Rattus1 | Vertebrate CRY1 |
|  |  |  | 1785 | NM_133405 | NP_596896 | Rattus2 | Vertebrate CRY2 |
|  |  | *Saimiri boliviensis boliviensis* | 1761 | XM_003929650 | XP_003929699 | Saimiri1 | Vertebrate CRY1 |
|  |  |  | 1782 | XM_003920024 | XP_003920073 | Saimiri2 | Vertebrate CRY2 |
|  |  | *Sorex araneus* | 1929 | XM_004619692 | XP_004619749 | Sorex1 | Vertebrate CRY1 |
|  |  |  | 1917 | XM_004619097 | XP_004619154 | Sorex2 | Vertebrate CRY2 |
|  |  | *Spermophilus tridecemlineatus* | 1764 | XM_005322323 | XP_005322380 | Spermo1 | Vertebrate CRY1 |
|  |  |  | 1782 | XM_005330030 | XP_005330087 | Spermo2 | Vertebrate CRY2 |
|  |  | *Sus scrofa* | 1767 | XM_003126079 | XP_003126127 | Sus1 | Vertebrate CRY1 |
|  |  |  | 1785 | XM_003122835 | XP_003122883 | Sus2 | Vertebrate CRY2 |
|  |  | *Tarsius syrichta* | 1647 | XM_008053581 | XP_008051772 | Tarsius1 | Vertebrate CRY1 |
|  |  |  | 1386 | XM_008050891 | XP_008049082 | Tarsius2 | Vertebrate CRY2 |
|  |  | *Trichechus manatus latirostris* | 1764 | XM_004373794 | XP_004373851 | Triche1 | Vertebrate CRY1 |
|  |  |  | 1782 | XM_004387653 | XP_004387710 | Triche2 | Vertebrate CRY2 |
|  |  | *Tupaia chinensis* | 1764 | XM_006170352 | XP_006170414 | Tupaia1 | Vertebrate CRY1 |
|  |  |  | 1623 | XM_006157843 | XP_006157905 | Tupaia2 | Vertebrate CRY2 |
|  |  | *Vicugna pacos* | 1764 | XM_006205864 | XP_006205926 | Vicugna1 | Vertebrate CRY1 |
|  |  |  | 1386 | XM_006212314 | XP_006212376 | Vicugna2 | Vertebrate CRY2 |
|  | Monotremata | *Ornithorhynchus anatinus* | 1824 | XM_007670116 | XP_007668306 | Ornithor1 | Vertebrate CRY1 |
|  |  |  | 1695 | XM_007668038 | XP_007666228 | Ornithor2 | Vertebrate CRY2 |
|  | Metatheria | *Monodelphis domestica* | 1764 | XM_007503484 | XP_007503546 | Monode1 | Vertebrate CRY1 |
|  |  |  | 1845 | XM_007497374 | XP_007497436 | Monode2 | Vertebrate CRY2 |
|  |  |  | 1413 | NM_001032977 | NP_001028149 | Monode_C | CPD class II PHR |
|  |  | *Sarcophilus harrisii* | 1857 | XM_003763671 | XP_003763719 | Sarcop_C | CPD class II PHR |
|  | Aves | *Anas platyrhynchos* | 1848 | XM_005011518 | XP_005011575 | Anas1 | Vertebrate CRY1 |
|  |  |  | 1383 | XM_005013969 | XP_005014026 | Anas2 | Vertebrate CRY2 |
|  |  |  | 1473 | XM_005027303 | XP_005027360 | Anas_d | CRY-DASH |
|  |  | *Columba livia* | 1812 | XM_005507610 | XP_005507667 | Columba1 | Vertebrate CRY1 |
|  |  |  | 1575 | XM_005502396 | XP_005502453 | Columba2 | Vertebrate CRY2 |
|  |  |  | 1578 | XM_005510337 | XP_005510394 | Columba4 | Vertebrate CRY4 |
|  |  |  | 1572 | XM_005509122 | XP_005509179 | Columba_d | CRY-DASH |
|  |  |  | 1512 | XM_005513231 | XP_005513288 | Columba_C | CPD class II PHR |
|  |  | *Falco peregrinus* | 1863 | XM_005237343 | XP_005237400 | Falco1 | Vertebrate CRY1 |
|  |  |  | 1569 | XM_005236380 | XP_005236437 | Falco2 | Vertebrate CRY2 |
|  |  |  | 1584 | XM_005239603 | XP_005239660 | Falco4 | Vertebrate CRY4 |
|  |  |  | 1539 | XM_005242928 | XP_005242985 | Falco_6 | (6-4) PHR |
|  |  |  | 1569 | XM_005240979 | XP_005241036 | Falco_d | CRY-DASH |
|  |  |  | 1509 | XM_005237811 | XP_005237868 | Falco_C | CPD class II PHR |
|  |  | *Ficedula albicollis* | 1863 | XM_005039847 | XP_005039904 | Ficedu1 | Vertebrate CRY1 |
|  |  |  | 1740 | XM_005047159 | XP_005047216 | Ficedu2 | Vertebrate CRY2 |
|  |  |  | 1584 | XM_005059308 | XP_005059365 | Ficedu4 | Vertebrate CRY4 |
|  |  |  | 1557 | XM_005040541 | XP_005040598 | Ficedu_d | CRY-DASH |
|  |  |  | 1506 | XM_005051145 | XP_005051202 | Ficedu_C | CPD class II PHR |
|  |  | *Gallus gallus* | 1866 | NM_204245 | NP_989576 | Gallus1 | Vertebrate CRY1 |
|  |  |  | 1749 | NM_204244 | NP_989575 | Gallus2 | Vertebrate CRY2 |
|  |  |  | 1590 | NM_001039596 | NP_001034685 | Gallus4 | Vertebrate CRY4 |
|  |  |  | 1500 | XM_422729 | XP_422729 | Gallus_C | CPD class II PHR |
|  |  | *Geospiza fortis* | 1794 | XM_005421906 | XP_005421963 | Geospi1 | Vertebrate CRY1 |
|  |  |  | 1611 | XM_005419552 | XP_005419609 | Geospi2 | Vertebrate CRY2 |
|  |  |  | 1584 | XM_005426815 | XP_005426872 | Geospi4 | Vertebrate CRY4 |
|  |  |  | 1467 | XM_005424356 | XP_005424413 | Geospi_d | CRY-DASH |
|  |  |  | 1425 | XM_005419108 | XP_005419165 | Geospi_C | CPD class II PHR |
|  |  | *Meleagris gallopavo* | 1836 | XM_003202363 | XP_003202411 | Meleag1 | Vertebrate CRY1 |
|  |  |  | 1623 | XM_003206441 | XP_003206489 | Meleag2 | Vertebrate CRY2 |
|  |  |  | 1593 | XM_003212851 | XP_003212899 | Meleag4 | Vertebrate CRY4 |
|  |  |  | 1512 | XM_003209143 | XP_003209191 | Meleag_C | CPD class II PHR |
|  |  | *Melopsittacus undulatus* | 1866 | XM_005150419 | XP_005150476 | Melopsi1 | Vertebrate CRY1 |
|  |  |  | 1692 | XM_005143964 | XP_005144021 | Melopsi2 | Vertebrate CRY2 |
|  |  |  | 1614 | XM_005150134 | XP_005150191 | Melopsi_d | CRY-DASH |
|  |  |  | 2733 | XM_005142335 | XP_005142392 | Melopsi_C | CPD class II PHR |
|  |  | *Pseudopodoces humilis* | 1863 | XM_005527535 | XP_005527592 | Pseudop1 | Vertebrate CRY1 |
|  |  |  | 1755 | XM_005522470 | XP_005522527 | Pseudop2 | Vertebrate CRY2 |
|  |  |  | 1584 | XM_005530203 | XP_005530260 | Pseudop4 | Vertebrate CRY4 |
|  |  |  | 1575 | XM_005527811 | XP_005527868 | Pseudop_d | CRY-DASH |
|  |  |  | 2022 | XM_005525174 | XP_005525231 | Pseudop_C | CPD class II PHR |
|  |  | *Taeniopygia guttata* | 1767 | XM_002196518 | XP_002196554 | Taeniopy1 | Vertebrate CRY1 |
|  |  |  | 1623 | XM_002198864 | XP_002198900 | Taeniopy2 | Vertebrate CRY2 |
|  |  |  | 1584 | XM_002198497 | XP_002198533 | Taeniopy4 | Vertebrate CRY4 |
|  |  |  | 1467 | XM_004176816 | XP_004176864 | Taeniopy_d | CRY-DASH |
|  |  |  | 2094 | XM_002190577 | XP_002190613 | Taeniopy_C | CPD class II PHR |
|  |  | *Zonotrichia albicollis* | 1863 | XM_005491170 | XP_005491227 | Zonotri1 | Vertebrate CRY1 |
|  |  |  | 1617 | XM_005480219 | XP_005480276 | Zonotri2 | Vertebrate CRY1 |
|  |  |  | 1697 | XM_005492438 | XP_005491511 | Zonotri4 | Vertebrate CRY4 |
|  |  |  | 1560 | XM_005491454 | XP_005491511 | Zonotri_d | CRY-DASH |
|  |  |  | 1506 | XM_005484901 | XP_005484958 | Zonotri_C | CPD class II PHR |
|  | Archosauria | *Alligator sinensis* | 1863 | XM_006024277 | XP_006024339 | Alliga1 | Vertebrate CRY1 |
|  |  |  | 1578 | XM_006026947 | XP_006027009 | Alliga2 | Vertebrate CRY2 |
|  |  |  | 1593 | XM_006021354 | XP_006021416 | Alliga_6 | (6-4) PHR |
|  |  |  | 1527 | XM_006022427 | XP_006022489 | Alliga_C | CPD class II PHR |
|  | Lepidosauria | *Anolis carolinensis* | 1866 | XM_003220922 | XP_003220970 | Anolis1 | Vertebrate CRY1 |
|  |  |  | 1761 | XM_003214641 | XP_003214689 | Anolis2 | Vertebrate CRY2 |
|  |  |  | 1593 | XM_008109915 | XP_008108122 | Anolis4 | Vertebrate CRY4 |
|  |  |  | 1593 | XM_008118246 | XP_008116453 | Anolis_6 | (6-4) PHR |
|  |  |  | 1665 | XM_008112202 | XP_008110409 | Anolis_d | CRY-DASH |
|  |  |  | 1608 | XM_003226963 | XP_003227011 | Anolis_C | CPD class II PHR |
|  |  | *Python bivittatus* | 1893 | XM_007432367 | XP_007432429 | Python1 | Vertebrate CRY1 |
|  |  |  | 1776 | XM_007436574 | XP_007436636 | Python2 | Vertebrate CRY2 |
|  |  |  | 1277 | XM_007441858 | XP_007441920 | Python_C | CPD class II PHR |
|  | Testudines | *Chelonia mydas* | 1863 | XM_007066141 | XP_007066203 | Chelon1 | Vertebrate CRY1 |
|  |  |  | 1455 | XM_007070839 | XP_007070901 | Chelon2 | Vertebrate CRY2 |
|  |  |  | 1602 | XM_007054312 | XP_007054374 | Chelon4 | Vertebrate CRY4 |
|  |  |  | 1593 | XM_007066377 | XP_007066439 | Chelon_6 | (6-4) PHR |
|  |  |  | 1611 | XM_007066561 | XP_007066623 | Chelon_d | CRY-DASH |
|  |  | *Chrysemys picta* | 1857 | XM_005300804 | XP_005300861 | Chryse1 | Vertebrate CRY1 |
|  |  |  | 1749 | XM_005302233 | XP_005302290 | Chryse2 | Vertebrate CRY2 |
|  |  |  | 1581 | XM_005294728 | XP_005294785 | Chryse_6 | (6-4) PHR |
|  |  |  | 1584 | XM_005297137 | XP_005297194 | Chryse_d | CRY-DASH |
|  |  |  | 1641 | XM_008173743 | XP_008171965 | Chryse_C | CPD class II PHR |
|  |  | *Pelodiscus sinensis* | 1923 | XM_006138065 | XP_006138127 | Pelodi1 | Vertebrate CRY1 |
|  |  |  | 1617 | XM_006124501 | XP_006124563 | Pelodi2 | Vertebrate CRY2 |
|  |  |  | 1602 | XM_006115696 | XP_006115758 | Pelodi4 | Vertebrate CRY4 |
|  |  |  | 1584 | XM_006120641 | XP_006120703 | Pelodi_d | CRY-DASH |
|  |  |  | 1773 | XM_006112378 | XP_006112440 | Pelodi_C | CPD class II PHR |
|  | Amphibia | *Xenopus tropicalis* | 1857 | NM_001017311 | NP_001017311 | Xenopus1 | Vertebrate CRY1 |
|  |  |  | 1989 | XM_002934092 | XP_002934138 | Xenopus2 | Vertebrate CRY2 |
|  |  |  | 1674 | NM_001130234 | NP_001123706 | Xenopus4 | Vertebrate CRY4 |
|  |  |  | 1596 | NM_001126540 | NP_001120012 | Xenopus_6 | (6-4) PHR |
|  |  |  | 1404 | XM_002938001 | XP_002938047 | Xenopus_d | CRY-DASH |
|  |  |  | 1464 | XM_004914834 | XP_004914891 | Xenopus_C | CPD class II PHR |
|  | Sarcopterygii | *Latimeria chalumnae* | 1962 | XM_005989252 | XP_005989314 | Latime1a | Vertebrate CRY1 |
|  |  |  | 1848 | XM_005987685 | XP_005987747 | Latime1b | Vertebrate CRY1 |
|  |  |  | 1185 | XM_005986968 | XP_005987030 | Latime2 | Vertebrate CRY2 |
|  | Actinopterygii | *Astyanax mexicanus* | 1878 | NM_001291262 | NP_001278191 | Astyan1a | Vertebrate CRY1 |
|  |  |  | 1674 | XM_007250531 | XP_007250593 | Astyan1b | Vertebrate CRY1 |
|  |  |  | 1434 | XM_007256752 | XP_007256814 | Astyan1c | Vertebrate CRY1 |
|  |  |  | 1629 | XM_007236111 | XP_007236173 | Astyan1d | Vertebrate CRY1 |
|  |  |  | 837 | XM_007237111 | XP_007237173 | Astyan2 | Vertebrate CRY2 |
|  |  |  | 1566 | XM_007244716 | XP_007244778 | Astyan_6 | (6-4) PHR |
|  |  | *Cynoglossus semilaevis* | 1896 | XM_008315395 | XP_008313617 | Cynoglo1a | Vertebrate CRY1 |
|  |  |  | 1956 | XM_008318937 | XP_008317159 | Cynoglo1b | Vertebrate CRY1 |
|  |  |  | 1698 | XM_008312353 | XP_008310575 | Cynoglo1c | Vertebrate CRY1 |
|  |  |  | 2007 | XM_008311224 | XP_008309446 | Cynoglo2 | Vertebrate CRY2 |
|  |  |  | 1569 | XM_008307928 | XP_00830615 | Cynoglo_d | CRY-DASH |
|  |  | *Danio rerio* | 1860 | NM_001077297 | NP_001070765 | Danio1a | Vertebrate CRY1 |
|  |  |  | 1821 | NM_131790 | NP_571865 | Danio1b | Vertebrate CRY1 |
|  |  |  | 1968 | NM_131791 | NP_571866 | Danio2a | Vertebrate CRY1 |
|  |  |  | 1917 | NM_131792 | NP_571867 | Danio2b | Vertebrate CRY1 |
|  |  |  | 1797 | NM_131786 | NP_571861 | Danio3 | Vertebrate CRY2 |
|  |  |  | 1677 | NM_131787 | NP_571862 | Danio4 | Vertebrate CRY4 |
|  |  |  | 1560 | NM_131788 | NP_571863 | Danio_6 | (6-4) PHR |
|  |  |  | 1563 | NM_205686 | NP_991249 | Danio_d | CRY-DASH |
|  |  |  | 1551 | NM_201064 | NP_957358 | Danio_C | CPD class II PHR |
|  |  | *Haplochromis burtoni* | 1893 | XM_005934221 | XP_005934283 | Haploch1a | Vertebrate CRY1 |
|  |  |  | 1713 | XM_005930296 | XP_005930358 | Haploch1b | Vertebrate CRY1 |
|  |  |  | 1815 | XM_005933227 | XP_005933289 | Haploch1c | Vertebrate CRY1 |
|  |  |  | 2022 | XM_005951184 | XP_005951246 | Haploch2 | Vertebrate CRY2 |
|  |  |  | 324 | XM_005952793 | XP_005952855 | Haploch2b | Vertebrate CRY2 |
|  |  |  | 1560 | XM_005934537 | XP_005934599 | Haploch_6 | (6-4) PHR |
|  |  |  | 1509 | XM_005912408 | XP_005912470 | Haploch_d | CRY-DASH |
|  |  |  | 1677 | XM_005939069 | XP_005939131 | Haploch_C | CPD class II PHR |
|  |  | *Lepisosteus oculatus* | 1860 | XM_006633084 | XP_006633147 | Lepiso1 | Vertebrate CRY1 |
|  |  |  | 1959 | XM_006628513 | XP_006628576 | Lepiso1b | Vertebrate CRY1 |
|  |  |  |  |  |  |  |  |
|  |  |  | 1716 | XM_006643120 | XP_006643183 | Lepiso2 | Vertebrate CRY2 |
|  |  |  | 1605 | XM_006642227 | XP_006642290 | Lepiso_6 | (6-4) PHR |
|  |  |  | 1572 | XM_006634261 | XP_006634324 | Lepiso_d | CRY-DASH |
|  |  |  | 1602 | XM_006637849 | XP_006637912 | Lepiso_C | CPD class II PHR |
|  |  | *Maylandia zebra* | 2010 | XM_004567610 | XP_004567667 | Mayland1a | Vertebrate CRY1 |
|  |  |  | 1815 | XM_004548441 | XP_004548498 | Mayland1b | Vertebrate CRY1 |
|  |  |  | 1713 | XM_004570794 | XP_004570851 | Mayland1c | Vertebrate CRY1 |
|  |  |  | 2022 | XM_004556204 | XP_004556261 | Mayland2 | Vertebrate CRY2 |
|  |  |  | 1557 | XM_004539582 | XP_004539639 | Mayland_6 | (6-4) PHR |
|  |  |  | 1566 | XM_004546757 | XP_004546814 | Mayland_d | CRY-DASH |
|  |  |  | 1674 | XM_004570480 | XP_004570537 | Mayland_C | CPD class II PHR |
|  |  | *Neolamprologus brichardi* | 1965 | XM_006807417 | XP_006807480 | Neolam1a | Vertebrate CRY1 |
|  |  |  | 1893 | XM_006780875 | XP_006780938 | Neolam1b | Vertebrate CRY1 |
|  |  |  | 1713 | XM_006795747 | XP_006795810 | Neolam1c | Vertebrate CRY1 |
|  |  |  | 2025 | XM_006782239 | XP_006782302 | Neolam2 | Vertebrate CRY2 |
|  |  |  | 1560 | XM_006779858 | XP_006779921 | Neolam_6 | (6-4) PHR |
|  |  |  | 1566 | XM_006795120 | XP_006795183 | Neolam_d | CRY-DASH |
|  |  | *Oreochromis niloticus* | 1893 | XM_005456618 | XP_005456675 | Oreo1a | Vertebrate CRY1 |
|  |  |  | 1956 | XM_005477745 | XP_005477802 | Oreo1b | Vertebrate CRY1 |
|  |  |  | 2019 | XM_003449249 | XP_003449297 | Oreo2 | Vertebrate CRY2 |
|  |  |  | 1569 | XM_003437598 | XP_003437646 | Oreoch_6 | (6-4) PHR |
|  |  |  | 1569 | XM_003437598 | XP_003437646 | Oreoch_6b | (6-4) PHR |
|  |  |  | 1566 | XM_003439198 | XP_003439246 | Oreoch_d | CRY-DASH |
|  |  |  | 1536 | XM_003438048 | XP_003438096 | Oreoch_C | CPD class II PHR |
|  |  | *Oryzias latipes* | 1875 | XM_004083204 | XP_004083252 | Oryzia1a | Vertebrate CRY1 |
|  |  |  | 1962 | XM_004086308 | XP_004086356 | Oryzia1b | Vertebrate CRY1 |
|  |  |  | 1689 | XM_004069867 | XP_004069915 | Oryzia1c | Vertebrate CRY1 |
|  |  |  | 2004 | XM_004070156 | XP_004070204 | Oryzia2 | Vertebrate CRY2 |
|  |  |  | 1566 | XM_004067403 | XP_004067451 | Oryzia_6 | (6-4) PHR |
|  |  |  | 1566 | XM_004081085 | XP_004081133 | Oryzia_d | CRY-DASH |
|  |  |  | 1515 | NM_001104801 | NP_001098271 | Oryzia_C | CPD class II PHR |
|  |  | *Poecilia formosa* | 1878 | XM_007555313 | XP_007555375 | Poecil1a | Vertebrate CRY1 |
|  |  |  | 1974 | XM_007566853 | XP_007566915 | Poecil1b | Vertebrate CRY1 |
|  |  |  | 1884 | XM_007561355 | XP_007561417 | Poecil1c | Vertebrate CRY1 |
|  |  |  | 1977 | XM_007567553 | XP_007567615 | Poecil2 | Vertebrate CRY2 |
|  |  |  | 852 | XM_007567529 | XP_007567591 | Poecil2b | Vertebrate CRY2 |
|  |  |  | 1566 | XM_007569237 | XP_007569299 | Poecil_6 | (6-4) PHR |
|  |  |  | 1566 | XM_007555551 | XP_007555613 | Poecil_d | CRY-DASH |
|  |  | *Pundamilia nyererei* | 1893 | XM_005747264 | XP_005747321 | Pundam1a | Vertebrate CRY1 |
|  |  |  | 1965 | XM_005750806 | XP_005750863 | Pundam1b | Vertebrate CRY1 |
|  |  |  | 1284 | XM_005734847 | XP_005734904 | Pundam1c | Vertebrate CRY1 |
|  |  |  | 1743 | XM_005747883 | XP_005747940 | Pundam2 | Vertebrate CRY2 |
|  |  |  | 1560 | XM_005733378 | XP_005733435 | Pundam_6 | (6-4) PHR |
|  |  |  | 1566 | XM_005722703 | XP_005722760 | Pundam_d | CRY-DASH |
|  |  |  | 1674 | XM_005752489 | XP_005752546 | Pundam_C | CPD class II PHR |
|  |  | *Stegastes partitus* | 1986 | XM_008283154 | XP_008281376 | Stega1a | Vertebrate CRY1 |
|  |  |  | 1878 | XM_008280885 | XP_008279107 | Stega1b | Vertebrate CRY1 |
|  |  |  | 1713 | XM_008290990 | XP_008289212 | Stega1c | Vertebrate CRY1 |
|  |  |  | 1878 | XM_008304928 | XP_008303150 | Stega2 | Vertebrate CRY2 |
|  |  |  | 1569 | XM_008278557 | XP_008276779 | Stega_6 | (6-4) PHR |
|  |  |  | 1566 | XM_008297727 | XP_008295949 | Stega_d | CRY-DASH |
|  |  |  | 1647 | XM_008285666 | XP_008283888 | Stega_C | CPD class II PHR |
|  |  | *Takifugu rubripes* | 2157 | XM_003976633 | XP_003976682 | Takif2 | Vertebrate CRY2 |
|  |  |  | 3966 | XM_003969514 | XP_003969563 | Takif_6 | (6-4) PHR |
|  |  |  | 1566 | XM_003967988 | XP_003968037 | Takif_d | CRY-DASH |
|  |  |  | 1617 | XM_003975795 | XP_003975844 | Takif_C | CPD class II PHR |
|  |  | *Xiphophorus maculatus* | 1974 | XM_005801889 | XP_005801946 | Xiphoph1a | Vertebrate CRY1 |
|  |  |  | 1878 | XM_005799961 | XP_005800018 | Xiphoph1b | Vertebrate CRY1 |
|  |  |  | 1725 | XM_005815648 | XP_005815705 | Xiphoph1c | Vertebrate CRY1 |
|  |  |  | 1971 | XM_005795894 | XP_005795951 | Xiphoph2 | Vertebrate CRY2 |
|  |  |  | 1566 | XM_005796202 | XP_005796259 | Xiphoph_6 | (6-4) PHR |
|  |  |  | 1575 | XM_005797823 | XP_005797880 | Xiphoph_d | CRY-DASH |
|  | Chondrichthyes | *Callorhinchus milii* | 1854 | XM_007895430 | XP_007893621 | Callor1 | Vertebrate CRY1 |
|  |  |  | 633 | XM_007911488 | XP_007909679 | Callor1b | Vertebrate CRY1 |
|  |  |  | 1716 | XM_007887759 | XP_007885950 | Callor2 | Vertebrate CRY2 |
|  | Cephalochordata | *Branchiostoma floridae* | 1542 | XM_002609455 | XP_002609501 | Branch1 | Vertebrate CRY |
|  |  |  | 1569 | XM_002609457 | XP_002609503 | Branch4 | Vertebrate CRY4 |
|  |  |  | 1524 | XM_002595028 | XP_002595074 | Branch4b | Vertebrate CRY4 |
| Echinodermata | | *Strongylocentrotus purpuratus* | 2265 | XM_780780 | XP_785873 | Strongy1 | Animal CRY |
|  |  |  | 1320 | XM_781238 | XP_786331 | Strongy1b | Animal CRY |
|  |  |  | 1575 | XM_783845 | XP_788938 | Strongy_6 | (6-4) PHR |
|  |  |  | 1254 | XM_778520 | XP_783613 | Strongy_d | CRY-DASH |
|  |  |  | 1506 | XM_003726260 | XP_003726308 | Strongy_C | CPD class II PHR |
| Arthropoda (Animalia) | Insecta | *Acyrthosiphon pisum* | 1662 | NM_001171061 | NP_001164532 | Acyrth1 | Insect CRY1 |
|  |  |  | 1539 | NM_001171102 | NP_001164573 | Acyrth2 | Insect CRY2 |
|  |  |  | 1608 | XM_001945977 | XP_001946012 | Acyrth_6 | (6-4) PHR |
|  |  |  | 1521 | XM_001949116 | XP_001949151 | Acyrth_C | CPD class II PHR |
|  |  | *Aedes aegypti* | 1638 | XM_001648448 | XP_001648498 | Aedes1 | Insect CRY1 |
|  |  |  | 2463 | XM_001655728 | XP_001655778 | Aedes2 | Insect CRY2 |
|  |  |  | 1662 | XM_001658145 | XP_001658195 | Aedes_6 | (6-4) PHR |
|  |  |  | 1521 | XM_001653905 | XP_001653955 | Aedes_C | CPD class II PHR |
|  |  | *Anopheles gambiae* | 1638 | XM_321104 | XP_321104 | Anoph1 | Insect CRY1 |
|  |  |  | 3171 | XM_313179 | XP_313179 | Anoph2 | Insect CRY2 |
|  |  |  | 1671 | XM_314748 | XP_314748 | Anoph_6 | (6-4) PHR |
|  |  |  | 1650 | XM_313925 | XP_313925 | Anoph_C | CPD class II PHR |
|  |  | *Apis mellifera* | 1713 | NM_001083630 | NP_001077099 | Apis2 | Insect CRY2 |
|  |  |  | 1443 | XM_003250426 | XP_003250474 | Apis_C | CPD class II PHR |
|  |  | *Bombus terrestris* | 1725 | XM_003398483 | XP_003398531 | Bombus2 | Insect CRY2 |
|  |  |  | 1485 | XM_003398046 | XP_003398094 | Bombus_C | CPD class II PHR |
|  |  | *Bombyx mori* | 1611 | NM_001195699 | NP_001182628 | Bombyx1 | Insect CRY1 |
|  |  |  | 2193 | NM_001195698 | NP_001182627 | Bombyx2 | Insect CRY2 |
|  |  | *Ceratitis capitata* | 1644 | XM_004529232 | XP_004529289 | Cerati1 | Insect CRY1 |
|  |  |  | 1605 | XM_004531238 | XP_004531295 | Cerati_6 | (6-4) PHR |
|  |  |  | 1572 | XM_004535674 | XP_004535731 | Cerati_C | CPD class II PHR |
|  |  | *Culex quinquefasciatus* | 1500 | XM_001851351 | XP_001851403 | Culex1 | Insect CRY1 |
|  |  |  | 2370 | XM_001865552 | XP_001865587 | Culex2a | Insect CRY2 |
|  |  |  | 2487 | XM_001869421 | XP_001869456 | Culex2b | Insect CRY2 |
|  |  |  | 1620 | XM_001867860 | XP_001867895 | Culex_6 | (6-4) PHR |
|  |  |  | 1533 | XM_001845605 | XP_001845657 | Culex_C | CPD class II PHR |
|  |  | *Drosophila melanogaster* | 1629 | NM_169852 | NP_732407 | Droso1 | Insect CRY1 |
|  |  |  | 1623 | NM_165334 | NP_724274 | Droso_6 | (6-4) PHR |
|  |  |  | 1668 | NM_078929 | NP_523653 | Droso_C | CPD class II PHR |
|  |  | *Megachile rotundata* | 1770 | XM_003707037 | XP_003707085 | Megachi2 | Insect CRY2 |
|  |  | *Musca domestica* | 1749 | XM_005178150 | XP_005178207 | Musca1 | Insect CRY1 |
|  |  |  | 1623 | XM_005182716 | XP_005182773 | Musca_6 | (6-4) PHR |
|  |  |  | 1560 | XM_005176998 | XP_005177055 | Musca_C | CPD class II PHR |
|  |  | *Nasonia vitripennis* | 1971 | XM_008206206 | XP_008204428 | Nasonia2 | Insect CRY2 |
|  |  |  | 1515 | XM_001603235 | XP_001603285 | Nasonia_C | CPD class II PHR |
|  |  | *Pediculus humanus corporis* | 1521 | XM_002430500 | XP_002430545 | Pediculus2 | Insect CRY2 |
|  |  | *Tribolium castaneum* | 1608 | NM_001083325 | NP_001076794 | Triboli2 | Insect CRY2 |
|  | Crustacea | *Daphnia pulex* | 1578 | GL732562 * | EFX77441 | DaphniaD | Insect CRY1 |
|  |  |  | 1662 | GL732540 * | EFX82092 | DaphniaM | Insect CRY2 |
|  |  |  | 1563 | GL732529 * | EFX86680 | Daphnia_d | CRY-DASH |
|  |  |  | 1575 | GL732532 * | EFX85418 | Daphnia_6 | (6-4) PHR |
|  |  |  | 1572 | GL732612 * | EFX71237 | Daphnia_C | CPD class II PHR |
| Mollusca (Animalia) | | *Aplysia californica* | 1686 | XM_005103876 | XP_005103933 | Aplysia1 | Animal CRY |
|  |  |  | 1632 | XM_005089685 | XP_005089742 | Aplysia1b | Animal CRY |
|  |  |  | 1581 | XM_005091008 | XP_005091065 | Aplysia_6 | (6-4) PHR |
|  |  |  | 1839 | XM_005098284 | XP_005098341 | Aplysia_d | CRY-DASH |
|  |  |  | 1662 | XM_005093253 | XP_005093310 | Aplysia_C | CPD class II PHR |
| Cnidaria (Animalia) | | *Hydra magnipapillata* | 1581 | XM_002166508 | XP_002166544 | Hydra_d | CRY-DASH |
|  |  | *Nematostella vectensis* | 1542 | XM_001632799 | XP_001632849 | Nemato1 | Animal CRY |
|  |  |  | 1479 | XM_001632800 | XP_001632850 | Nemato1b | Animal CRY |
|  |  |  | 1626 | XM_001623096 | XP_001623146 | Nemato1c | Animal CRY |
|  |  |  | 1392 | XM_001625330 | XP_001625380 | Nemato1d | Animal CRY |
|  |  |  | 1602 | XM_001636253 | XP_001636303 | Nemato_6 | (6-4) PHR |
|  |  |  | 1575 | XM_001623193 | XP_001623243 | Nemato_d | CRY-DASH |
|  |  |  | 1149 | XM_001636204 | XP_001636254 | Nemato_C | CPD class II PHR |
|  |  |  | 1101 | XM_001620382 | XP_001620432 | Nemato_C2 | CPD class II PHR |
| Nematoda (Animalia) | | *Brugia malayi* | 1329 | XM_001902257 | XP_001902292 | Brugia_C | CPD class II PHR |
|  |  | *Loa loa* | 786 | XM_003146941 | XP_003146989 | Loa_C | CPD class II PHR |
|  |  | *Trichinella spiralis* | 1548 | XM_003381288 | XP_003381336 | Trichin_C | CPD class II PHR |
| Euglenozoa | | *Leishmania major* | 1626 | XM_001685734 | XP_001685786 | Leish_C | CPD class I PHR |
|  |  | *Trypanosoma brucei* | 1707 | XM_818226 | XP_823319 | Trypano_C | CPD class I PHR |
| Amoebozoa | | *Acanthamoeba castellanii* | 1734 | XM_004341074 | XP_004341122 | Acantha_C | CPD class II PHR |
| Heterolobosea | | *Naegleria gruberi* | 1662 | XM_002670658 | XP_002670704 | Naegler_d | CRY-DASH |
| Alveolata | Ciliophora | *Tetrahymena thermophila* | 1461 | XM_001014668 | XP_001014668 | Tetrahy_C | CPD class I PHR |
|  |  |  | 1419 | XM_001015321 | XP_001015321 | Tetrahy_C2 | CPD class I PHR |
|  | Perkinsea | *Perkinsus marinus* | 1443 | XM_002773825 | XP_002773871 | Perkin_C | CPD class II PHR |
|  | Apicomplexa | *Plasmodium vivax* | 2922 | XM_001613770 | XP_001613820 | Plasmo_C | CPD class II PHR |
|  |  | *Toxoplasma gondii* | 1896 | XM_002367780 | XP_002367821 | Toxopla_C | CPD class II PHR |
| Stramenopiles | Bacillariophyta | *Phaeodactylum tricornutum* | 1653 | XM_002180059 | XP_002180095 | Phaeoda_6 | (6-4) PHR |
|  |  |  | 1833 | XM_002178853 | XP_002178889 | Phaeoda_d | CRY-DASH |
|  |  |  | 1534 | XM_002180035 | XP_002180071 | Phaeoda_C | CPD class II PHR |
|  |  | *Thalassiosira pseudonana* | 1670 | XM_002291108 | XP_002291144 | Thalassi_6 | (6-4) PHR |
|  |  |  | 1716 | XM_002291289 | XP_002291325 | Thalassi_d | CRY-DASH |
|  |  |  | 1407 | XM_002290995 | XP_002291031 | Thalassi_C | CPD class II PHR |
|  | Oomycetes | *Phytophthora infestans* | 1707 | XM_002908292 | XP_002908338 | Phytoph_6 | (6-4) PHR |
|  |  |  | 1749 | XM_002895233 | XP_002895279 | Phytoph_C | CPD class II PHR |
|  | Eustigmatophyceae | *Nannochloropsis gaditana* | 1683 | XM_005852575 | XP_005852637 | Nannoch_6 | (6-4) PHR |
|  |  |  | 1887 | AZIL01000287 | EWM28467 | Nannoch_d | CRY-DASH |
| Rhodophyta | | *Chondrus crispus* | 1863 | XM_005711341 | XP_005711398 | Chondr_d | CRY-DASH |
|  |  | *Cyanidioschyzon merolae* | 1797 | XM_005534989 | XP_005535046 | Cyanidi_d | CRY-DASH |
| Cryptophyta | | *Guillardia theta* | 1482 | XM_005838359 | XP_005838416 | Guillar_d | CRY-DASH |
| Choanoflagellates | | *Monosiga brevicollis* | 1461 | XM_001747454 | XP_001747506 | Monosi_6 | (6-4) PHR |
|  |  |  | 1578 | XM_001745105 | XP_001745157 | Monosi_d | CRY-DASH |
|  |  |  | 2655 | XM_001746614 | XP_001746666 | Monosi_C | CPD class II PHR |
|  |  | *Salpingoeca* sp. ATCC 50818 | 1659 | XM_004988951 | XP_004989008 | Salpin_d | CRY-DASH |
| Viridiplantae | Liliopsida | *Brachypodium distachyon* | 2139 | XM_003579826 | XP_003579874 | Brachypo1a | Plant CRY1 |
|  |  |  | 2121 | XM_003575157 | XP_003575205 | Brachypo1b | Plant CRY1 |
|  |  |  | 1914 | XM_003575345 | XP_003575393 | Brachypo2 | Plant CRY2 |
|  |  |  | 1806 | XM_003571470 | XP_003571518 | Brachypo_6 | (6-4) PHR |
|  |  |  | 1764 | XM_003563301 | XP_003563349 | Brachypo_d | CRY-DASH |
|  |  |  | 1512 | XM_003573760 | XP_003573808 | Brachypo_C | CPD class II PHR |
|  |  |  | 1344 | XM_003557892 | XP_003557940 | Brac_PHR2 | Plant PHR2 |
|  |  | *Oryza sativa* | 2157 | NM_001053735 | NP_001047200 | Oryza1a | Plant CRY1 |
|  |  |  | 2103 | NM_001059485 | NP_001052950 | Oryza1b | Plant CRY1 |
|  |  |  | 1005 | NM_001186153 | NP_001173082 | Oryza2 | Plant CRY2 |
|  |  |  | 1656 | NM_001052778 | NP_001046243 | Oryza_6 | (6-4) PHR |
|  |  |  | 1644 | NM_001064813 | NP_001058278 | Oryza_d | CRY-DASH |
|  |  |  | 1446 | AB210109 | BAE45635 | Oryza_C | CPD class II PHR |
|  |  |  | 1380 | NM_001056612 | NP_001050077 | Oryz_PHR2 | Plant PHR2 |
|  |  | *Setaria italica* | 2151 | XM_004975751 | XP_004975808 | Setaria1a | Plant CRY1 |
|  |  |  | 2121 | XM_004952796 | XP_004952853 | Setaria1b | Plant CRY1 |
|  |  |  | 1929 | XM_004965357 | XP_004965414 | Setaria2 | Plant CRY2 |
|  |  |  | 1728 | XM_004951456 | XP_004951513 | Setaria_6 | (6-4) PHR |
|  |  |  | 1824 | XM_004976886 | XP_004976943 | Setaria_6b | (6-4) PHR |
|  |  |  | 1746 | XM_004965588 | XP_004965645 | Setaria_d | CRY-DASH |
|  |  |  | 1500 | XM_004983271 | XP_004983328 | Setaria_C | CPD class II PHR |
|  |  |  | 1341 | XM_004984373 | XP_004984430 | Seta_PHR2 | Plant PHR2 |
|  |  | *Sorghum bicolor* | 1965 | XM_002436943 | XP_002436988 | Sorghum2 | Plant CRY2 |
|  |  |  | 1653 | XM_002451702 | XP_002451747 | Sorghum_6 | (6-4) PHR |
|  |  |  | 1734 | XM_002438751 | XP_002438796 | Sorghum_d | CRY-DASH |
|  |  |  | 1344 | XM_002467834 | XP_002467879 | Sorg_PHR2 | Plant PHR2 |
|  |  | *Zea mays* | 2100 | NM_001177006 | NP_001170477 | Zea1 | Plant CRY1 |
|  |  |  | 1932 | NM_001197008 | NP_001183937 | Zea1b | Plant CRY1 |
|  |  |  | 1287 | NM_001139179 | NP_001132651 | Zea2 | Plant CRY2 |
|  |  |  | 1653 | NM_001153235 | NP_001146707 | Zea_d | CRY-DASH |
|  |  |  | 1482 | NM_001137108 | NP_001130580 | Zea_C | CPD class II PHR |
|  |  |  | 1341 | NM_001137536 | NP_001131008 | Zea_PHR2 | Plant PHR2 |
|  | Eudicotidae | *Arabidopsis thaliana* | 2046 | NM_116961 | NP_567341 | Arabi1 | Plant CRY1 |
|  |  |  | 1839 | NM_100320 | NP_171935 | Arabi2 | Plant CRY2 |
|  |  |  | 1671 | NM_112432 | NP_566520 | Arabi_6 | (6-4) PHR |
|  |  |  | 1338 | NM_001035626 | NP_001030703 | Arabi_6b | (6-4) PHR |
|  |  |  | 1581 | NM_122394 | NP_568461 | Arabi_d | CRY-DASH |
|  |  |  | 1491 | NM_179320 | NP_849651 | Arabi_C | CPD class II PHR |
|  |  |  | 1473 | NM_101109 | NP_563906 | Arabi_C2 | CPD class II PHR |
|  |  |  | 1344 | NM_130327 | NP_182281 | Arab_PHR2 | Plant PHR2 |
|  |  | *Capsella rubella* | 1578 | XM_006287429 | XP_006287491 | Capsella1a | Plant CRY1 |
|  |  |  | 1545 | XM_006287428 | XP_006287490 | Capsella1 | Plant CRY1 |
|  |  |  | 1839 | XM_006306962 | XP_006307024 | Capsella2 | Plant CRY2 |
|  |  |  | 1677 | XM_006299297 | XP_006299359 | Capsella_6 | (6-4) PHR |
|  |  |  | 1704 | XM_006287323 | XP_006287385 | Capsella_d | CRY-DASH |
|  |  |  | 1467 | XM_006306070 | XP_006306132 | Capsella_C | CPD class II PHR |
|  |  |  | 1536 | XM_006293972 | XP_006294034 | Caps_PHR2 | Plant PHR2 |
|  |  | *Cicer arietinum* | 2046 | XM_004490318 | XP_004490375 | Cicer1 | Plant CRY1 |
|  |  |  | 1932 | XM_004495325 | XP_004495382 | Cicer2a | Plant CRY2 |
|  |  |  | 1953 | XM_004497786 | XP_004497843 | Cicer2b | Plant CRY2 |
|  |  |  | 1632 | XM_004506833 | XP_004506890 | Cicer_6 | (6-4) PHR |
|  |  |  | 1776 | XM_004501196 | XP_004501253 | Cicer_d | CRY-DASH |
|  |  |  | 1491 | XM_004511588 | XP_004511645 | Cicer_C | CPD class II PHR |
|  |  |  | 1380 | XM_004495888 | XP_004495945 | Cice_PHR2 | Plant PHR2 |
|  |  | *Citrus clementina* | 2046 | XM_006433921 | XP_006433984 | Citrus1 | Plant CRY1 |
|  |  |  | 1935 | XM_006420156 | XP_006420219 | Citrus2 | Plant CRY2 |
|  |  |  | 1632 | XM_006427120 | XP_006427183 | Citrus_6 | (6-4) PHR |
|  |  |  | 1317 | XM_006450063 | XP_006450126 | Citrus_d | CRY-DASH |
|  |  |  | 1479 | XM_006440356 | XP_006440419 | Citrus_C | CPD class II PHR |
|  |  |  | 1398 | XM_006444307 | XP_006444370 | Citr_PHR2 | Plant PHR2 |
|  |  | *Cucumis sativus* | 2046 | XM_004160230 | XP_004160278 | Cucumis1a | Plant CRY1 |
|  |  |  | 2046 | XM_004136242 | XP_004136290 | Cucumis1b | Plant CRY1 |
|  |  |  | 1635 | XM_004158759 | XP_004158807 | Cucumis_6 | (6-4) PHR |
|  |  |  | 1650 | XM_004136032 | XP_004136080 | Cucumis_6b | (6-4) PHR |
|  |  |  | 1650 | XM_004136273 | XP_004136321 | Cucumis_d | CRY-DASH |
|  |  |  | 1470 | NM_001280653 | NP_001267582 | Cucumis_C | CPD class II PHR |
|  |  |  | 1380 | XM_004135976 | XP_004136024 | Cucu_PHR2 | Plant PHR2 |
|  |  | *Eutrema salsugineum* | 1674 | XM_006406881 | XP_006406944 | Eutrema_6 | (6-4) PHR |
|  |  |  | 1650 | XM_006394675 | XP_006394737 | Eutrema_d | CRY-DASH |
|  |  |  | 1488 | XM_006417179 | XP_006417242 | Eutrema_C | CPD class II PHR |
|  |  |  | 1350 | XM_006397890 | XP_006397953 | Eutr_PHR2 | Plant PHR2 |
|  |  | *Fragaria vesca* | 2022 | NM_001280031 | NP_001266960 | Fragaria1 | Plant CRY1 |
|  |  |  | 1938 | NM_001280040 | NP_001266969 | Fragaria2 | Plant CRY2 |
|  |  |  | 1647 | XM_004302889 | XP_004302937 | Fragaria_6 | (6-4) PHR |
|  |  |  | 1584 | XM_004305554 | XP_004305602 | Fragaria_d | CRY-DASH |
|  |  |  | 1383 | XM_004290753 | XP_004290801 | Frag_PHR2 | Plant PHR2 |
|  |  | *Glycine max* | 2049 | NM_001254073 | NP_001241002 | Glycine1 | Plant CRY1 |
|  |  |  | 2046 | NM_001248276 | NP_001235205 | Glycine1a | Plant CRY1 |
|  |  |  | 2046 | NM_001255223 | NP_001242152 | Glycine1b | Plant CRY1 |
|  |  |  | 2046 | NM_001253926 | NP_001240855 | Glycine1c | Plant CRY1 |
|  |  |  | 1905 | NM_001289325 | NP_001276254 | Glycine2 | Plant CRY2 |
|  |  |  | 1905 | NM_001248291 | NP_001235220 | Glycine2a | Plant CRY2 |
|  |  |  | 1803 | NM_001254622 | NP_001241551 | Glycine2b | Plant CRY2 |
|  |  |  | 1692 | XM_003531652 | XP_003531700 | Glycine_6 | (6-4) PHR |
|  |  |  | 1650 | XM_003522608 | XP_003522656 | Glycine_d | CRY-DASH |
|  |  |  | 1503 | NM_001251781 | NP_001238710 | Glycine_C | CPD class II PHR |
|  |  |  | 1323 | XM_003536065 | XP_003536113 | Glyc_PHR2 | Plant PHR2 |
|  |  |  | 1287 | XM_003518945 | XP_003518993 | Glyc_PHR2b | Plant PHR2 |
|  |  | *Medicago truncatula* | 2040 | XM_003615064 | XP_003615112 | Medica1 | Plant CRY1 |
|  |  |  | 1803 | XM_003589988 | XP_003590036 | Medica2 | Plant CRY2 |
|  |  |  | 1101 | XM_003603475 | XP_003603523 | Medica_d | CRY-DASH |
|  |  |  | 1554 | XM_003611189 | XP_003611237 | Medica_C | CPD class II PHR |
|  |  |  | 1371 | XM_003591466 | XP_003591514 | Medi_PHR2 | Plant PHR2 |
|  |  | *Phaseolus vulgaris* | 2048 | XM_007141118 | XP_007141180 | Phaseo1 | Plant CRY1 |
|  |  |  | 2025 | XM_007136191 | XP_007136253 | Phaseo1b | Plant CRY1 |
|  |  |  | 1755 | XM_007136192 | XP_007136254 | Phaseo1c | Plant CRY1 |
|  |  |  | 1908 | XM_007143808 | XP_007143870 | Phaseo2a | Plant CRY2 |
|  |  |  | 1890 | XM_007145785 | XP_007145847 | Phaseo2b | Plant CRY2 |
|  |  |  | 1797 | XM_007145786 | XP_007145848 | Phaseo2c | Plant CRY2 |
|  |  |  | 1611 | XM_007135665 | XP_007135727 | Phaseo_6 | (6-4) PHR |
|  |  |  | 1776 | XM_007137046 | XP_007137108 | Phaseo_d | CRY-DASH |
|  |  |  | 1506 | XM_007157254 | XP_007157316 | Phaseo_C | CPD class II PHR |
|  |  |  | 1353 | XM_007144269 | XP_007144331 | Phas_PHR2 | Plant PHR2 |
|  |  | *Populus trichocarpa* | 2043 | XM_002307343 | XP_002307379 | Populus1 | Plant CRY1 |
|  |  |  | 1758 | XM_006648709 | XP_006378337 | Populus2 | Plant CRY2 |
|  |  |  | 1614 | XM_002312545 | XP_002312581 | Populus2b | Plant CRY2 |
|  |  |  | 1623 | XM_002304127 | XP_002304163 | Populus_6 | (6-4) PHR |
|  |  |  | 1602 | XM_006385429 | XP_006385491 | Populus_6b | (6-4) PHR |
|  |  |  | 1704 | XM_006382131 | XP_006382193 | Populus_d | CRY-DASH |
|  |  |  | 1494 | XM_006368651 | XP_006368713 | Populus_C | CPD class II PHR |
|  |  |  | 1416 | XM_002302680 | XP_002302716 | Popu_PHR2 | Plant PHR2 |
|  |  |  | 945 | XM_006375392 | XP_006375454 | Popu_PHR2b | Plant PHR2 |
|  |  | *Prunus mume* | 2040 | XM_008221215 | XP_008219437 | Prunus1 | Plant CRY1 |
|  |  |  | 1941 | XM_008226137 | XP_008224359 | Prunus2 | Plant PHR2 |
|  |  |  | 1614 | XM_008230419 | XP_008228641 | Prunus_6 | (6-4) PHR |
|  |  |  | 1752 | XM_008221586 | XP_008219808 | Prunus_d | CRY-DASH |
|  |  |  | 1500 | XM_008240430 | XP_008238652 | Prunus_C | CPD class II PHR |
|  |  |  | 1380 | XM_008236881 | XP_008235103 | Prun_PHR2 | Plant PHR2 |
|  |  | *Ricinus communis* | 1983 | XM_002513876 | XP_002513922 | Ricinus1 | Plant CRY1 |
|  |  |  | 1923 | XM_002531483 | XP_002531529 | Ricinus2 | Plant CRY2 |
|  |  |  | 1614 | XM_002517528 | XP_002517574 | Ricinus_6 | (6-4) PHR |
|  |  |  | 1731 | XM_002527929 | XP_002527975 | Ricinus_d | CRY-DASH |
|  |  |  | 1737 | XM_002527930 | XP_002527976 | Ricinus_d2 | CRY-DASH |
|  |  |  | 1470 | XM_002509575 | XP_002509621 | Ricinus_C | CPD class II PHR |
|  |  |  | 1377 | XM_002523046 | XP_002523092 | Rici_PHR2 | Plant PHR2 |
|  |  | *Solanum lycopersicum* | 2040 | NM_001247738 | NP_001234667 | Solanum1 | Plant CRY1 |
|  |  |  | 1752 | NM_001247648 | NP_001234577 | Solanum1b | Plant CRY1 |
|  |  |  | 1908 | NM_001247316 | NP_001234245 | Solanum2 | Plant CRY2 |
|  |  |  | 1620 | XM_004252275 | XP_004252323 | Solanum_6 | (6-4) PHR |
|  |  |  | 1734 | XM_004246030 | XP_004246078 | Solanum_d | CRY-DASH |
|  |  |  | 1467 | XM_004245655 | XP_004245703 | Solanum_C | CPD class II PHR |
|  |  |  | 1359 | XM_004247365 | XP_004247413 | Sola_PHR2 | Plant PHR2 |
|  |  | *Theobroma cacao* | 2049 | XM_007018710 | XP_007018772 | Theobro1 | Plant CRY1 |
|  |  |  | 1932 | XM_007035047 | XP_007035109 | Theobro2 | Plant CRY2 |
|  |  |  | 1554 | XM_007023877 | XP_007023939 | Theobro_6 | (6-4) PHR |
|  |  |  | 1749 | XM_007011490 | XP_007011552 | Theobro_d | CRY-DASH |
|  |  |  | 1488 | XM_007039927 | XP_007039989 | Theobro_C | CPD class II PHR |
|  |  |  | 1401 | XM_007050882 | XP_007050944 | Theo_PHR2 | Plant PHR2 |
|  |  | *Vitis vinifera* | 2046 | XM_002285756 | XP_002285792 | Vitis1 | Plant CRY1 |
|  |  |  | 1941 | XM_002285133 | XP_002285169 | Vitis2 | Plant CRY2 |
|  |  |  | 1617 | XM_002285291 | XP_002285327 | Vitis_6 | (6-4) PHR |
|  |  |  | 1650 | XM_002280735 | XP_002280771 | Vitis_d | CRY-DASH |
|  |  |  | 1458 | XM_002266447 | XP_002266483 | Vitis_C | CPD class II PHR |
|  |  |  | 1365 | XM_002270212 | XP_002270248 | Viti_PHR2 | Plant PHR2 |
|  | Amborellales | *Amborella trichopoda* | 1602 | XM_006853095 | XP_006853157 | Ambore1 | Plant CRY1 |
|  |  |  | 1953 | XM_006846768 | XP_006846831 | Ambore2 | Plant CRY2 |
|  |  |  | 1614 | XM_006841838 | XP_006841901 | Ambore_6 | (6-4) PHR |
|  |  |  | 1662 | XM_006850840 | XP_006850903 | Ambore_d | CRY-DASH |
|  |  |  | 1473 | XM_006851787 | XP_006851849 | Ambore_C | CPD class II PHR |
|  |  |  | 1401 | XM_006854409 | XP_006854471 | Ambo_PHR2 | Plant PHR2 |
|  | Lycopodiophyta | *Selaginella moellendorffii* | 1482 | XM_002961206 | XP_002961252 | Selagin1a | Plant CRY |
|  |  |  | 1482 | XM_002969440 | XP_002969486 | Selagin1b | Plant CRY |
|  |  |  | 1620 | XM_002964128 | XP_002964174 | Selagin2a | Plant CRY |
|  |  |  | 1467 | XM_002988844 | XP_002988890 | Selagin2b | Plant CRY |
|  |  |  | 1680 | XM_002969868 | XP_002969914 | Selagin3a | Plant CRY |
|  |  |  | 1680 | XM_002981345 | XP_002981391 | Selagin3b | Plant CRY |
|  |  |  | 1584 | XM_002979587 | XP_002979633 | Selagin_6 | (6-4) PHR |
|  |  |  | 1302 | XM_002977595 | XP_002977641 | Selagin_6b | (6-4) PHR |
|  |  |  | 1500 | XM_002976861 | XP_002976907 | Selagin_d1 | CRY-DASH |
|  |  |  | 1500 | XM_002980581 | XP_002980627 | Selagin_d2 | CRY-DASH |
|  |  |  | 1533 | XM_002971995 | XP_002972041 | Selagin_C | CPD class II PHR |
|  |  |  | 1533 | XM_002972541 | XP_002972587 | Selagin_C2 | CPD class II PHR |
|  |  |  | 1038 | XM_002966213 | XP_002966259 | Sela_PHR2 | Plant PHR2 |
|  |  |  | 1038 | XM_002978267 | XP_002978313 | Sela_PHR2b | Plant PHR2 |
|  | Bryophyta | *Physcomitrella patens* | 2184 | XM_001751711 | XP_001751763 | Physco1a | Plant Cry |
|  |  |  | 1308 | XM_001751901 | XP_001751953 | Physco1b | Plant Cry |
|  |  |  | 1581 | XM_001754022 | XP_001754074 | Physco_6 | (6-4) PHR |
|  |  |  | 1674 | XM_001785534 | XP_001785586 | Physco_d | CRY-DASH |
|  |  |  | 1488 | XM_001764938 | XP_001764990 | Physco_C | CPD class II PHR |
|  |  |  | 1587 | XM_001760511 | XP_001760563 | Phys_PHR2 | Plant PHR2 |
|  | Chlorophyta | *Bathycoccus prasinos* | 1677 | XM_007509236 | XP_007509298 | Bathyco_d | CRY-DASH |
|  |  | *Chlamydomonas reinhardtii* | 3027 | XM_001701501 | XP_001701553 | Chlamy1 | Plant CRY |
|  |  |  | 1788 | XM_001698002 | XP_001698054 | Chlamy_6 | (6-4) PHR |
|  |  |  | 1407 | XM_001701819 | XP_001701871 | Chlamy_d1 | CRY-DASH |
|  |  |  | 1943 | XM_001690000 | XP_001690052 | Chlamy_d2 | CRY-DASH |
|  |  |  | 2841 | XM_001692854 | XP_001692906 | Chlamy_C | Putative (6-4) PHR |
|  |  |  | 1331 | XM_001692944 | XP_001692996 | Chla_PHR2 | Plant PHR2 |
|  |  | *Chlorella variabilis* | 1473 | XM_005843416 | XP_005843478 | Chlorella_6 | (6-4) PHR |
|  |  |  | 3555 | XM_005848261 | XP_005848323 | Chlorella_d | CRY-DASH |
|  |  |  | 1335 | XM_005851029 | XP_005851091 | Chlorella_C | CPD class II PHR |
|  |  | *Coccomyxa subellipsoidea* | 1407 | XM_005646919 | XP_005646976 | Coccomy1 | Plant CRY |
|  |  |  | 1449 | XM_005646741 | XP_005646798 | Coccomy_6 | (6-4) PHR |
|  |  |  | 1701 | XM_005646668 | XP_005646725 | Coccomy_d | CRY-DASH |
|  |  | *Micromonas pusilla* | 1680 | XM_003061182 | XP_003061228 | Microm_6 | (6-4) PHR |
|  |  |  | 1686 | XM_003062934 | XP_003062980 | Microm_d | CRY-DASH |
|  |  | *Micromonas* sp. RCC299 | 1740 | XM_002507137 | XP_002507183 | Microm_C | CPD class II PHR |
|  |  | *Ostreococcus lucimarinus* | 1656 | XM_001415659 | XP_001415696 | Ostreoc_d | CRY-DASH |
|  |  |  | 1521 | XM_001417058 | XP_001417095 | Ostreoc_C | CPD class II PHR |
|  |  | *Volvox carteri* | 2745 | XM_002957226 | XP_002957272 | Volvox1 | Plant CRY |
|  |  |  | 1866 | XM_002945880 | XP_002945926 | Volvox_6 | (6-4) PHR |
|  |  |  | 3078 | XM_002953647 | XP_002953693 | Volvox_d | CRY-DASH |
|  |  |  | 1770 | XM_002953258 | XP_002953304 | Volvox_C | CPD class II PHR |
|  |  |  | 1866 | XM_002945880 | XP_002945926 | Volvox_6 | (6-4) PHR |
| Fungi | Ascomycota | *Arthroderma gypseum* | 1770 | XM_003172684 | XP_003172732 | Arthro_C | CPD class I PHR |
|  |  | *Aspergillus clavatus* | 1842 | XM_001270767 | XP_001270768 | Asperg_6 | (6-4) PHR |
|  |  |  | 1761 | XM_001269978 | XP_001269979 | Asperg_C | CRY-DASH |
|  |  | *Baudoinia compniacensis* | 1944 | XM_007673580 | XP_007671770 | Baudoin_6 | (6-4) PHR |
|  |  |  | 1737 | XM_007679948 | XP_007678138 | Baudoin_d | CRY-DASH |
|  |  |  | 1800 | XM_007674890 | XP_007673080 | Baudoin_C | CPD class I PHR |
|  |  |  | 1650 | XM_007676468 | XP_007674658 | Baudoin_C2 | CPD class I PHR |
|  |  | *Bipolaris zeicola* | 1962 | XM_007719059 | XP_007717249 | Bipolar_6 | (6-4) PHR |
|  |  |  | 2046 | XM_007719672 | XP_007717862 | Bipolar_d | CRY-DASH |
|  |  |  | 1905 | XM_007715814 | XP_007714004 | Bipolar_C | CPD class I PHR |
|  |  |  | 1605 | XM_007716773 | XP_007714963 | Bipolar_C2 | CPD class I PHR |
|  |  | *Botryotinia fuckeliana* | 1956 | XM_001553265 | XP_001553315 | Botryo_d | CRY-DASH |
|  |  |  | 1824 | XM_001548392 | XP_001548442 | Botryo_C | CPD class I PHR |
|  |  | *Candida tenuis* | 1626 | XM_006688111 | XP_006688174 | Candida_C | CPD class I PHR |
|  |  | *Capronia coronata* | 2925 | XM_007726825 | XP_007725015 | Capron_6 | (6-4) PHR |
|  |  |  | 1791 | XM_007733528 | XP_007731718 | Capron_d | CRY-DASH |
|  |  |  | 1743 | XM_007727531 | XP_007725721 | Capron_C | CPD class I PHR |
|  |  |  | 1638 | XM_007724564 | XP_007722754 | Capron_C2 | CPD class I PHR |
|  |  | *Cladophialophora yegresii* | 2187 | XM_007754749 | XP_007752939 | Cladoph_6 | (6-4) PHR |
|  |  |  | 1890 | XM_007757487 | XP_007755677 | Cladoph_d | CRY-DASH |
|  |  |  | 1764 | XM_007755973 | XP_007754163 | Cladoph_C | CPD class I PHR |
|  |  |  | 1629 | XM_007759544 | XP_007757734 | Cladoph_C2 | CPD class I PHR |
|  |  | *Clavispora lusitaniae* | 1647 | XM_002614411 | XP_002614457 | Clavisp_C | CPD class I PHR |
|  |  | *Cochliobolus sativus* | 1965 | XM_007705700 | XP_007703890 | Cochlio_6 | (6-4) PHR |
|  |  |  | 2022 | XM_007695988 | XP_007694178 | Cochlio_d | CRY-DASH |
|  |  |  | 2623 | XM_007701575 | XP_007699765 | Cochlio_C | CPD class I PHR |
|  |  |  | 1608 | XM_007700300 | XP_007698490 | Cochlio_C2 | CPD class I PHR |
|  |  | *Colletotrichum fioriniae* | 1884 | XM_007590147 | XP_007590209 | Collet_6 | (6-4) PHR |
|  |  |  | 2148 | XM_007596031 | XP_007596093 | Collet_d | CRY-DASH |
|  |  |  | 2013 | XM_007593545 | XP_007593607 | Collet_C | CPD class I PHR |
|  |  | *Cordyceps militaris* | 2709 | XM_006667544 | XP_006667607 | Cordyce_6 | (6-4) PHR |
|  |  |  | 1974 | XM_006665933 | XP_006665996 | Cordyce_d | CRY-DASH |
|  |  |  | 2319 | XM_006665311 | XP_006665374 | Cordyce_C | CPD class I PHR |
|  |  | *Debaryomyces hansenii* | 1668 | XM_456362 | XP_456362 | Debaryo_C | CPD class I PHR |
|  |  | *Endocarpon pusillum* | 1578 | XM_007803106 | XP_007801297 | Endocar_d | CRY-DASH |
|  |  |  | 1794 | XM_007805291 | XP_007803482 | Endocar_C | CPD class I PHR |
|  |  |  | 1605 | XM_007788277 | XP_007786467 | Endocar_C2 | CPD class I PHR |
|  |  | *Eutypa lata* | 1863 | XM_007790411 | XP_007788602 | Eutypa_6 | (6-4) PHR |
|  |  |  | 1926 | XM_007797974 | XP_007796165 | Eutypa_d | CRY-DASH |
|  |  |  | 1857 | XM_007791079 | XP_007789270 | Eutypa_C | CPD class I PHR |
|  |  | *Gibberella zeae* | 2058 | XM_386941 | XP_386941 | Gibber_6 | (6-4) PHR |
|  |  |  | 2037 | XM_389028 | XP_389028 | Gibber_d | CRY-DASH |
|  |  |  | 1908 | XM_380973 | XP_380973 | Gibber_C | CPD class I PHR |
|  |  | *Glarea lozoyensis* | 1917 | XM_008082279 | XP_008080470 | Glarea_6 | (6-4) PHR |
|  |  |  | 2052 | XM_008088869 | XP_008087060 | Glarea_d | CRY-DASH |
|  |  |  | 1806 | XM_008090340 | XP_008088531 | Glarea_C | CPD class I PHR |
|  |  | *Kazachstania africana* | 1737 | XM_003956564 | XP_003956613 | Kazach_C | CPD class I PHR |
|  |  | *Kluyveromyces lactis* | 1788 | XM_453092 | XP_453092 | Kluyver_C | CPD class I PHR |
|  |  | *Lachancea thermotolerans* | 1725 | XM_002556260 | XP_002556306 | Lachan_C | CPD class I PHR |
|  |  |  | 1725 | XM_002556260 | XP_002556306 | Lachan_C2 | CPD class I PHR |
|  |  | *Leptosphaeria maculans* | 2130 | XM_003835034 | XP_003835082 | Leptos_6 | (6-4) PHR |
|  |  |  | 2970 | XM_003836362 | XP_003836410 | Leptos_d | CRY-DASH |
|  |  |  | 2436 | XM_003834653 | XP_003834701 | Leptos_C | CPD class I PHR |
|  |  |  | 1902 | XM_003838353 | XP_003838401 | Leptos_C2 | CPD class I PHR |
|  |  | *Magnaporthe oryzae* | 2040 | XM_003708788 | XP_003708836 | Magnap_6 | (6-4) PHR |
|  |  |  | 2481 | XM_003720642 | XP_003720690 | Magnap_d | CRY-DASH |
|  |  |  | 1890 | XM_003709504 | XP_003709552 | Magnap_C | CPD class I PHR |
|  |  | *Marssonina brunnea* | 1953 | XM_007289971 | XP_007290033 | Marsson_6 | (6-4) PHR |
|  |  |  | 1983 | XM_007295026 | XP_007295088 | Marsson_d | CRY-DASH |
|  |  |  | 1905 | XM_007291766 | XP_007291828 | Marsson_C | CPD class I PHR |
|  |  | *Metarhizium acridum* | 1815 | XM_007811852 | XP_007810043 | Metarh_6 | (6-4) PHR |
|  |  |  | 2088 | XM_007821768 | XP_007819959 | Metarh_d | CRY-DASH |
|  |  |  | 1764 | XM_007813640 | XP_007811831 | Metarh_C | CPD class I PHR |
|  |  | *Meyerozyma guilliermondii* | 1824 | XM_001482016 | XP_001482066 | Meyero_C | CPD class I PHR |
|  |  | *Millerozyma farinosa* | 1680 | XM_004204673 | XP_004204721 | Millero_C | CPD class I PHR |
|  |  | *Mycosphaerella graminicola* | 2013 | XM_003857321 | XP_003857369 | Mycosph_6 | (6-4) PHR |
|  |  |  | 1710 | XM_003853533 | XP_003853581 | Mycosph_d | CRY-DASH |
|  |  |  | 1779 | XM_003857724 | XP_003857772 | Mycosph_C | CPD class I PHR |
|  |  |  | 1668 | XM_003847546 | XP_003847594 | Mycosph_C2 | CPD class I PHR |
|  |  | *Nectria* *haematococca* | 1920 | XM_003051746 | XP_003051792 | Nectria_6 | (6-4) PHR |
|  |  |  | 1920 | XM_003052722 | XP_003052768 | Nectria_d | CRY-DASH |
|  |  |  | 1794 | XM_003053813 | XP_003053859 | Nectria_C | CPD class I PHR |
|  |  | *Neofusicoccum parvum* | 1887 | XM_007579744 | XP_007579806 | Neofusic_6 | (6-4) PHR |
|  |  |  | 1635 | XM_007585929 | XP_007585991 | Neofusic_d | CRY-DASH |
|  |  |  | 1833 | XM_007584040 | XP_007584102 | Neofusic_C | CPD class I PHR |
|  |  | *Neosartorya fischeri* | 1755 | XM_001265491 | XP_001265492 | Neosar_C | CPD class I PHR |
|  |  | *Neurospora crassa* | 2169 | XM_960629 | XP_965722 | Neuros_d | CRY-DASH |
|  |  |  | 1929 | XM_959741 | XP_964834 | Neuros_C | CPD class I PHR |
|  |  | *Penicillium chrysogenum* | 1746 | XM_002566103 | XP_002566149 | Penici_C | CPD class I PHR |
|  |  | *Pestalotiopsis fici* | 1884 | XM_007841038 | XP_007839229 | Pestalo_6 | (6-4) PHR |
|  |  |  | 2046 | XM_007838442 | XP_007836633 | Pestalo_d | CRY-DASH |
|  |  |  | 1866 | XM_007831095 | XP_007829286 | Pestalo_C | CPD class I PHR |
|  |  |  | 1647 | XM_007836563 | XP_007834754 | Pestalo_C2 | CPD class I PHR |
|  |  | *Phaeosphaeria nodorum* | 3690 | XM_001801873 | XP_001801925 | Phaeosph_6 | (6-4) PHR |
|  |  |  | 1749 | XM_001797992 | XP_001798044 | Phaeosph_d | CRY-DASH |
|  |  |  | 1899 | XM_001791155 | XP_001791207 | Phaeosph_C | CPD class I PHR |
|  |  |  | 1605 | XM_001790929 | XP_001790981 | Phaeosph_C2 | CPD class I PHR |
|  |  | *Podospora anserina* | 2442 | XM_001906929 | XP_001906964 | Podosp_d | CRY-DASH |
|  |  |  | 2205 | XM_001912232 | XP_001912267 | Podosp_C | CPD class I PHR |
|  |  | *Pseudocercospora fijiensis* | 1968 | XM_007923821 | XP_007922012 | Pseudoce_6 | (6-4) PHR |
|  |  |  | 1836 | XM_007924063 | XP_007922254 | Pseudoce _d | CRY-DASH |
|  |  |  | 1770 | XM_007927647 | XP_007925838 | Pseudoce_C | CPD class I PHR |
|  |  |  | 1614 | XM_007924210 | XP_007922401 | Pseudoce_C2 | CPD class I PHR |
|  |  | *Pyrenophora tritici-repentis* | 1968 | XM_001930429 | XP_001930464 | Pyreno_6 | (6-4) PHR |
|  |  |  | 2028 | XM_001936447 | XP_001936482 | Pyreno_d | CRY-DASH |
|  |  |  | 1899 | XM_001941034 | XP_001941069 | Pyreno_C | CPD class I PHR |
|  |  |  | 1605 | XM_001937559 | XP_001937594 | Pyreno_C2 | CPD class I PHR |
|  |  | *Saccharomyces cerevisiae* | 1698 | NM_001183806 | NP_015031 | Saccha_C | CPD class I PHR |
|  |  | *Sclerotinia sclerotiorum* | 1956 | XM_001593685 | XP_001593735 | Sclero_d | CRY-DASH |
|  |  |  | 1788 | XM_001586997 | XP_001587047 | Sclero_C | CPD class I PHR |
|  |  | *Setosphaeria turcica* | 1977 | XM_008023807 | XP_008021998 | Setosph_6 | (6-4) PHR |
|  |  |  | 2055 | XM_008023394 | XP_008021585 | Setosph_d | CRY-DASH |
|  |  |  | 1905 | XM_008027057 | XP_008025248 | Setosph_C | CPD class I PHR |
|  |  |  | 1605 | XM_008028505 | XP_008026696 | Setosph_C2 | CPD class I PHR |
|  |  | *Sordaria macrospora* | 1593 | XM_003352392 | XP_003352440 | Sordar_d | CRY-DASH |
|  |  |  | 1665 | XM_003347554 | XP_003347602 | Sordar_C | CPD class I PHR |
|  |  | *Talaromyces stipitatus* | 1782 | XM_002480821 | XP_002480866 | Talarom_C | CPD class I PHR |
|  |  | *Togninia minima* | 1914 | XM_007914022 | XP_007912213 | Togni_6 | (6-4) PHR |
|  |  |  | 1590 | XM_007915434 | XP_007913625 | Togni_d | CRY-DASH |
|  |  |  | 1770 | XM_007921129 | XP_007919320 | Togni_C | CPD class I PHR |
|  |  | *Torulaspora delbrueckii* | 1695 | XM_003678513 | XP_003678561 | Torula_C | CPD class I PHR |
|  |  | *Trichoderma reesei* | 1887 | XM_006964865 | XP_006964927 | Trichode_6 | (6-4) PHR |
|  |  |  | 1665 | XM_006964290 | XP_006964352 | Trichode_d | CRY-DASH |
|  |  |  |  |  |  |  |  |
|  |  |  | 1776 | XM_006965711 | XP_006965773 | Trichode_C | CPD class I PHR |
|  |  | *Trichophyton rubrum* | 1770 | XM_003237443 | XP_003237491 | Tricho_C | CPD class I PHR |
|  |  | *Verticillium albo-atrum* | 1881 | XM_003006382 | XP_003006428 | Vertici_6 | (6-4) PHR |
|  |  |  | 2109 | XM_003008977 | XP_003009023 | Vertici_d | CRY-DASH |
|  |  |  | 1770 | XM_002999887 | XP_002999933 | Vertici_C | CPD class I PHR |
|  |  | *Zygosaccharomyces rouxii* | 1749 | XM_002494901 | XP_002494946 | Zygosa_C | CPD class I PHR |
|  |  |  | 1674 | XM_002498905 | XP_002498950 | Zygosa_C2 | CPD class I PHR |
|  | Basidiomycota | *Agaricus bisporus* | 1689 | XM_006460176 | XP_006460239 | Agaric_C | CPD class I PHR |
|  |  | *Auricularia delicata* | 1410 | XM_007345634 | XP_007345696 | Auricu_d | CRY-DASH |
|  |  |  | 1779 | XM_007338009 | XM_007338009 | Auricu_C | CPD class I PHR |
|  |  | *Coniophora puteana* | 1977 | XM_007774452 | XP_007772642 | Conioph_6 | (6-4) PHR |
|  |  |  | 1689 | XM_007769157 | XP_007767347 | Conioph_C | CPD class I PHR |
|  |  | *Coprinopsis cinerea* | 1902 | XM_002911174 | XP_002911220 | Coprino_C | CPD class I PHR |
|  |  | *Dichomitus squalens* | 1767 | XM_007365938 | XP_007366000 | Dicho_C | CPD class I PHR |
|  |  | *Fomitiporia mediterranea* | 1527 | XM_007267770 | XP_007267832 | Fomitip_d | CRY-DASH |
|  |  |  | 3168 | XM_007268445 | XP_007268507 | Fomitip_C | CPD class I PHR |
|  |  | *Gloeophyllum trabeum* | 1602 | XM_007863463 | XP_007861654 | Gloeoph_C | CPD class I PHR |
|  |  | *Melampsora larici-populina* | 1737 | XM_007407030 | XP_007407092 | Melamp_C | CPD class I PHR |
|  |  | *Moniliophthora roreri* | 1821 | XM_007846215 | XP_007844406 | Monilio_6 | (6-4) PHR |
|  |  |  | 1776 | XM_007849825 | XP_007848016 | Monilio_d | CRY-DASH |
|  |  |  | 3114 | XM_007848012 | XP_007846203 | Monilio_C | CPD class I PHR |
|  |  | *Phanerochaete carnosa* | 1608 | XM_007392279 | XP_007392341 | Phanero_C | CPD class I PHR |
|  |  | *Postia placenta* | 1632 | XM_002470069 | XP_002470114 | Postia_C | CPD class I PHR |
|  |  | *Pseudozyma flocculosa* | 2100 | XM_007877618 | XP_007875809 | Psudoz_6 | (6-4) PHR |
|  |  |  | 1878 | XM_007880104 | XP_007878295 | Psudoz_d | CRY-DASH |
|  |  |  | 2160 | XM_007879552 | XP_007877743 | Psudoz_C | CPD class I PHR |
|  |  | *Puccinia graminis* | 2196 | XM_003336176 | XP_003336224 | Puccinia_6 | (6-4) PHR |
|  |  |  | 1791 | XM_003326582 | XP_003326630 | Puccinia_d | CRY-DASH |
|  |  |  | 1827 | XM_003324053 | XP_003324101 | Puccinia_C | CPD class I PHR |
|  |  | *Punctularia strigosozonata* | 1587 | XM_007381864 | XP_007381926 | Punctu_C | CPD class I PHR |
|  |  | *Schizophyllum commune* | 1764 | XM_003036489 | XP_003036535 | Schizo_C | CPD class I PHR |
|  |  | *Serpula lacrymans* | 2051 | XM_007315847 | XP_007315909 | Serpu_C | CPD class I PHR |
|  |  | *Stereum hirsutum* | 1890 | XM_007300654 | XP_007300716 | Stereum_6 | (6-4) PHR |
|  |  |  | 1926 | XM_007303133 | XP_007303195 | Stereum_d | CRY-DASH |
|  |  |  | 1680 | XM_007298852 | XP_007298914 | Stereum_C | CPD class I PHR |
|  |  | *Tremella mesenterica* | 1644 | XM_007005908 | XP_007005970 | Treme_6 | (6-4) PHR |
|  |  |  | 1986 | XM_007006597 | XP_007006659 | Treme_d | CRY-DASH |
|  |  |  | 1644 | XM_007007641 | XP_007007703 | Treme_C | CPD class I PHR |
|  |  | *Trametes versicolor* | 1719 | XM_008036886 | XP_008035077 | Tramet_C | CPD class I PHR |
|  |  | *Ustilago maydis* | 2055 | XM_753198 | XP_758291 | Ustilago_6 | (6-4) PHR |
|  |  |  | 1872 | XM_756971 | XP_762064 | Ustilago_d | CRY-DASH |
|  |  |  | 1968 | XM_757133 | XP_762226 | Ustilago_C | CPD class I PHR |
|  |  | *Wallemia sebi* | 1665 | XM_006957275 | XP_006957337 | Wallem_6 | (6-4) PHR |
|  |  |  | 1635 | XM_006958808 | XP_006958870 | Wallem_C | CPD class I PHR |
|  | Microsporidia | *Nosema ceranae* | 1422 | XM_002996583 | XP_002996629 | Nosema_C | CPD class II PHR |
| Archaea | | *Halorhabdus utahensis* | 1437 | NC_013158 | YP_003131490 | Halorh_d | CRY-DASH |
|  |  |  | 1413 | NC_013158 | YP_003131773 | Halorh_C | CPD class I PHR |
|  |  | *Methanosarcina barkeri* | 1389 | NC_007355 | YP_304088 | Methano_C | CPD class II PHR |
| Bacteria | | *Cronobacter sakazakii* | 1422 | NC_009778 | YP_001438714 | Cronob_C | CPD class I PHR |
|  |  | *Geobacter sulfurreducens* | 1386 | NC_002939 | NP_953872 | Geoba_C | CPD class II PHR |
|  |  | *Gloeobacter violaceus* | 1473 | NC_005125 | NP_924695 | Gloeob_6 | (6-4) PHR |
|  |  | *Spirosoma linguale* | 1464 | NC_013730 | YP_003390944 | Spiros_d | CRY-DASH |

* DNA sequences are not available
