# Supplementary material for: Evolutionary History of the Photolyase/Cryptochrome Superfamily in Eukaryotes
Source: PLoS One. 2015 Sep 9;10(9):e0135940. doi: 10.1371/journal.pone.0135940 (PMC4564169; doi:10.1371/journal.pone.0135940)
Supplement: S2 Table — (DOCX) [file pone.0135940.s004.docx]

#### S2 Table – List of RPB2 sequences used in the study.

| Groups | | Species and taxa | Length (bp) | GenBank nucleotide sequence accession number | Protein accession number | Designation of sequence in figure |
| --- | --- | --- | --- | --- | --- | --- |
| Chordata (Animalia) |  | *Ailuropoda melanoleuca ** | 3504 | GL192578 | EFB28083 | Ailuro |
|  |  | *Bos taurus* | 3525 | NM_001099082 | NP_001092552 | Bos |
|  |  | *Camelus ferus* | 3255 | KB017425 | EPY77710 | Camelus |
|  |  | *Canis lupus familiaris* | 3978 | XM_532382 | XP_532382 | Canis |
|  |  | *Ceratotherium simum simum* | 3261 | XM_004419092 | XP_004419149 | Cerato |
|  |  | *Chrysochloris asiatica* | 3171 | XM_006868069 | XP_006868131 | Chrysoch |
|  |  | *Condylura cristata* | 3525 | XM_004681272 | XP_004681329 | Condy |
|  |  | *Cricetulus griseus* | 3243 | KE663574 | ERE91274 | Cricetu |
|  |  | *Dasypus novemcinctus* | 3525 | XM_004483866 | XP_004483923 | Dasypus |
|  |  | *Echinops telfairi* | 3525 | XM_004706294 | XP_004706351 | Echinops |
|  |  | *Elephantulus edwardii* | 3525 | XM_006891966 | XP_006892028 | Eleph |
|  | Eutheria | *Eptesicus fuscus* | 3300 | XM_008140336 | XP_008138558 | Eptesi |
|  |  | *Equus caballus* | 3525 | XM_001916791 | XP_001916826 | Equus |
|  |  | *Gorilla gorilla gorilla* | 3294 | XM_004038720 | XP_004038768 | Gorilla |
|  |  | *Heterocephalus glaber* | 3525 | XM_004910250 | XP_004910307 | Hetero |
|  |  | *Homo sapiens* | 3525 | NM_000938 | NP_000929 | Homo |
|  |  | *Jaculus jaculus* | 3432 | XM_004665333 | XP_004665390 | Jaculus |
|  |  | *Loxodonta africana* | 3033 | XM_003415932 | XP_003415980 | Loxo |
|  |  | *Macaca fascicularis* | 3369 | XM_005555208 | XP_005555265 | Macaca |
|  |  | *Mesocricetus auratus* | 3300 | XM_005080754 | XP_005080811 | Mesocri |
|  |  | *Mus musculus* | 3525 | NM_153798 | NP_722493 | Mus |
|  |  | *Myotis lucifugus* | 3549 | XM_006093932 | XP_006093994 | Myotis |
|  |  | *Ochotona princeps* | 3504 | XM_004590863 | [XP_004590920](http://www.ncbi.nlm.nih.gov/protein/504159205) | Ochotona |
|  |  | *Odobenus rosmarus* | 3261 | XM_004394954 | XP_004395011 | Odoben |
|  |  | *Orycteropus afer* | 3204 | XM_007946229 | XP_007944420 | Orycte |
|  |  | *Oryctolagus cuniculus* | 3525 | XM_002717160 | XP_002717206 | Orycto |
|  |  | *Pan troglodytes* | 3681 | XM_003806498 | XP_003806546 | Pan |
|  |  | *Papio anubis* | 3252 | XM_003898847 | XP_003898896 | Papio |
|  |  | *Pongo abelii* | 3504 | XM_002814754 | XP_002814800 | Pongo |
|  |  | *Pteropus alecto* | 3525 | XM_006922527 | XP_006922589 | Pteropus |
|  |  | *Saimiri boliviensis boliviensis* | 2979 | XM_003933667 | XP_003933716 | Saimiri |
|  |  | *Sorex araneus* | 2913 | XM_004616461 | XP_004616518 | Sorex |
|  |  | *Sus scrofa* | 3756 | XM_003129037 | XP_003129085 | Sus |
|  |  | *Trichechus manatus latirostris* | 3525 | XM_004382877 | XP_004382934 | Triche |
|  |  | *Tupaia chinensis* | 3525 | XM_006167707 | XP_006167769 | Tupaia |
|  | Monotremata | *Ornithorhynchus anatinus* | 2538 | XM_007664350 | [XP_007662540](http://www.ncbi.nlm.nih.gov/protein/620972320) | Ornithor |
|  | Metatheria | *Monodelphis domestica* | 3525 | XM_001362418 | XP_001362455 | Monode |
|  | Aves | *Anas platyrhynchos* | 3789 | XM_005028892 | XP_005028949 | Anas |
|  |  | *Columba livia ** | 3507 | KB375788 | EMC81333 | Columba |
|  |  | *Falco peregrinus* | 3504 | XM_005232517 | XP_005232574 | Falco |
|  |  | *Ficedula albicollis* | 3504 | XM_005044802 | XP_005044859 | Ficedu |
|  |  | *Gallus gallus* | 3525 | NM_001006448 | NP_001006448 | Gallus |
|  |  | *Geospiza fortis* | 3471 | XM_005418220 | XP_005418277 | Geospi |
|  |  | *Melopsittacus undulatus* | 3525 | XM_005148965 | XP_005149022 | Melopsi |
|  |  | *Taeniopygia guttata* | 3525 | XM_002192182 | XP_002192218 | Taeniopy |
|  | Archosauria | *Alligator sinensis* | 3534 | XM_006027763 | XP_006027825 | Alliga |
|  | Lepidosauria | *Anolis carolinensis* | 3525 | XM_003224643 | XP_003224691 | Anolis |
|  |  | *Python bivittatus* | 3525 | XM_007427010 | XP_007427072 | Python |
|  | Testudines | *Chelonia mydas* | 3504 | XM_007066464 | XP_007066526 | Chelon |
|  |  | *Chrysemys picta* | 3525 | XM_008162783 | XP_008161005 | Chryse |
|  |  | *Pelodiscus sinensis* | 3480 | XM_006127979 | XP_006128041 | Pelodi |
|  | Amphibia | *Xenopus tropicalis* | 3525 | XM_002937552 | XP_002937598 | Xenopus |
|  | Sarcopterygii | *Latimeria chalumnae* | 3531 | XM_006011843 | XP_006011905 | Latime |
|  | Actinopterygii | *Astyanax mexicanus* | 3243 | XM_007260334 | XP_007260396 | Astyan |
|  |  | *Cynoglossus semilaevis* | 3525 | XM_008326790 | XP_008325012 | Cynoglo |
|  |  | *Danio rerio* | 3525 | NM_001024461 | NP_001019632 | Danio |
|  |  | *Haplochromis burtoni* | 3543 | XM_005948655 | XP_005948717 | Haploch |
|  |  | *Lepisosteus oculatus* | 3525 | XM_006629739 | XP_006629802 | Lepiso |
|  |  | *Maylandia zebra* | 3642 | XM_004563633 | XP_004563690 | Mayland |
|  |  | *Neolamprologus brichardi* | 3525 | XM_006781925 | XP_006781988 | Neolam |
|  |  | *Oreochromis niloticus* | 3525 | XM_003458660 | XP_003458708 | Oreo |
|  |  | *Oryzias latipes* | 3363 | XM_004086548 | XP_004086596 | Oryzia |
|  |  | *Poecilia formosa* | 3525 | XM_007542803 | XP_007542865 | Poecil |
|  |  | *Pundamilia nyererei* | 3558 | XM_005719379 | XP_005719436 | Pundam |
|  |  | *Stegastes partitus* | 2307 | XM_008303822 | XP_008302044 | Stega |
|  |  | *Takifugu rubripes* | 2949 | XM_003979379 | XP_003979428 | Takif |
|  |  | *Xiphophorus maculatus* | 3450 | XM_005812296 | XP_005812353 | Xiphoph |
|  | Chondrichthyes | *Callorhinchus milii* | 3525 | XM_007891944 | XP_007890135 | Callor |
|  | Cephalochordata | *Branchiostoma floridae* | 3315 | XM_002608290 | XP_002608336 | Branch |
|  | Tunicata | *Ciona intestinalis* | 3522 | XM_002128880 | XP_002128916 | Ciona |
| Echinodermata (Animalia) | | *Strongylocentrotus purpuratus* | 3429 | XM_003724522 | XP_003724570 | Strongy |
| Arthropoda (Animalia) | Insecta | *Acyrthosiphon pisum* | 3534 | XM_008185197 | XP_008183419 | Acyrth |
|  |  | *Aedes aegypti ** | 3531 | CH477265 | EJY57456 | Aedes |
|  |  | *Anopheles gambiae* | 3528 | XM_313416 | XP_313416 | Anoph |
|  |  | *Apis mellifera* | 3468 | XM_006558332 | XP_006558395 | Apis |
|  |  | *Bombus terrestris* | 3528 | XM_003401429 | XP_003401477 | Bombus |
|  |  | *Bombyx mori* | 3528 | XM_004930876 | XP_004930933 | Bombyx |
|  |  | *Ceratitis capitata* | 3531 | XM_004534092 | XP_004534149 | Cerati |
|  |  | *Culex quinquefasciatus* | 3531 | XM_001849131 | XP_001849183 | Culex |
|  |  | *Drosophila melanogaster* | 3531 | NM_057358 | NP_476706 | Droso |
|  |  | *Megachile rotundata* | 3528 | XM_003701324 | XP_003701372 | Megachi |
|  |  | *Musca domestica* | 3531 | XM_005188782 | XP_005188839 | Musca |
|  |  | *Nasonia vitripennis* | 3765 | XM_008209300 | XP_008207522 | Nasonia |
|  |  | *Pediculus humanus corporis* | 3528 | XM_002425468 | XP_002425513 | Pediculus |
|  |  | *Tribolium castaneum* | 3528 | XM_969560 | XP_974653 | Triboli |
|  | Crustacea | *Daphnia pulex ** | 3525 | GL732545 | EFX81055 | Daphnia |
| Mollusca (Animalia) | | *Aplysia californica* | 3540 | XM_005104862 | XP_005104919 | Aplysia |
| Cnidaria (Animalia) | | *Hydra magnipapillata* | 3555 | XM_002163473 | XP_002163509 | Hydra |
|  |  | *Nematostella vectensis* | 3549 | XM_001628910 | XP_001628960 | Nemato |
| Nematoda (Animalia) | | *Brugia malayi* | 3558 | XM_001900527 | XP_001900562 | Brugia |
|  |  | *Loa loa* | 3516 | XM_003142143 | XP_003142191 | Loa |
|  |  | *Trichinella spiralis* | 3573 | XM_003374284 | XP_003374332 | Trichin |
| Euglenozoa | | *Leishmania major* | 3558 | XM_001684948 | XP_001685000 | Leish |
|  |  | *Trypanosoma brucei* | 3573 | XM_839438 | XP_844531 | Trypano |
| Amoebozoa | | *Acanthamoeba castellanii* | 3591 | XM_004348480 | XP_004348530 | Acantha |
| Heterolobosea | | *Naegleria gruberi* | 3702 | XM_002669567 | XP_002669613 | Naegler |
| Alveolata | Ciliophora | *Tetrahymena thermophila* | 3681 | XM_001015664 | XP_001015664 | Tetrahy |
|  | Perkinsea | *Perkinsus marinus* | 3516 | XM_002766842 | XP_002766888 | Perkin |
|  | Apicomplexa | *Toxoplasma gondii* | 3780 | XM_002365004 | XP_002365045 | Toxopla |
| Stramenopiles | Bacillariophyta | *Phaeodactylum tricornutum* | 3639 | XM_002178890 | XP_002178926 | Phaeoda |
|  |  | *Thalassiosira pseudonana* | 3591 | XM_002291327 | XP_002291363 | Thalassi |
|  | Oomycetes | *Phytophthora infestans* | 3609 | XM_002899993 | XP_002900039 | Phytoph |
|  | Eustigmatophyceae | *Nannochloropsis gaditana* | 3609 | AZIL01000179 | EWM29030 | Nannoch |
| Rhodophyta | | *Chondrus crispus* | 3423 | XM_005717944 | XP_005718001 | Chondr |
|  |  | *Cyanidioschyzon merolae* | 3555 | XM_005536023 | XP_005536080 | Cyanidi |
| Cryptophyta | | *Guillardia theta* | 3558 | XM_005836511 | XP_005836568 | Guillar |
| Choanoflagellates | | *Monosiga brevicollis* | 3501 | XM_001744227 | XP_001744279 | Monosi |
|  |  | *Salpingoeca* sp. ATCC 50818 | 3582 | XM_004996721 | XP_004996778 | Salpin |
| Angiosperms (Viridiplantae) | Liliopsida | *Brachypodium distachyon* | 3687 | XM_003561825 | XP_003561873 | Brachypo |
|  |  | *Oryza sativa ** | 3666 | DP000009 | ABF97884 | Oryza |
|  |  | *Setaria italica* | 3681 | XM_004982259 | XP_004982316 | Setaria |
|  |  | *Sorghum bicolor* | 3657 | XM_002466705 | XP_002466750 | Sorghum |
|  |  | *Zea mays ** | 2286 | GK000031 | DAA50302 | Zea |
|  | Eudicotidae | *Arabidopsis thaliana* | 3567 | NM_118291 | NP_193902 | Arabi |
|  |  | *Capsella rubella* | 3567 | XM_006285515 | XP_006285577 | Capsella |
|  |  | *Cicer arietinum* | 3585 | XM_004499160 | XP_004499217 | Cicer |
|  |  | *Citrus clementina* | 3588 | XM_006423531 | XP_006423594 | Citrus |
|  |  | *Cucumis sativus* | 3600 | XM_004134657 | XP_004134705 | Cucumis |
|  |  | *Eutrema salsugineum* | 3567 | XM_006413680 | XP_006413743 | Eutrema |
|  |  | *Fragaria vesca* | 3576 | XM_004293974 | XP_004294022 | Fragaria |
|  |  | *Glycine max* | 3582 | XM_003547434 | XP_003547482 | Glycine |
|  |  | *Medicago truncatula* | 3642 | XM_003589324 | XP_003589372 | Medica |
|  |  | *Phaseolus vulgaris* | 3585 | XM_007148106 | XP_007148168 | Phaseo |
|  |  | *Populus trichocarpa* | 3648 | XM_002305100 | XP_002305136 | Populus |
|  |  | *Prunus mume* | 3579 | XM_008226870 | XP_008225092 | Prunus |
|  |  | *Ricinus communis* | 3585 | XM_002519014 | XP_002519060 | Ricinus |
|  |  | *Solanum lycopersicum* | 3576 | NM_001246960 | NP_001233889 | Solanum |
|  |  | *Theobroma cacao* | 3570 | XM_007025569 | XP_007025631 | Theobro |
|  |  | *Vitis vinifera* | 3582 | XM_002274015 | XP_002274051 | Vitis |
|  | Amborellales | *Amborella trichopoda* | 3573 | XM_006858976 | XP_006859038 | Ambore |
| Lycopodiophyta (Viridiplantae) | | *Selaginella moellendorffii* | 3537 | XM_002972317 | XP_002972363 | Selagin |
| Bryophyta (Viridiplantae) | | *Physcomitrella patens* | 3558 | XM_001766373 | XP_001766425 | Physco |
| Chlorophyta (Viridiplantae) | | *Chlamydomonas reinhardtii* | 4509 | XM_001701782 | XP_001701834 | Chlamy |
|  |  | *Chlorella variabilis* | 3528 | XM_005844227 | XP_005844289 | Chlorella |
|  |  | *Coccomyxa subellipsoidea* | 3513 | XM_005643426 | XP_005643483 | Coccomy |
|  |  | *Micromonas* sp. RCC299 | 3537 | XM_002500494 | XP_002500540 | Microm |
|  |  | *Ostreococcus lucimarinus* | 3525 | XM_001415410 | XP_001415447 | Ostreoc |
|  |  | *Volvox carteri* | 3621 | XM_002947643 | XP_002947689 | Volvox |
| Fungi | Ascomycota | *Arthroderma gypseum* | 3777 | XM_003170096 | XP_003170144 | Arthro |
|  |  | *Aspergillus clavatus* | 3774 | XM_001272354 | XP_001272355 | Asperg |
|  |  | *Baudoinia compniacensis* | 3765 | XM_007675569 | XP_007673759 | Baudoin |
|  |  | *Botryotinia fuckeliana* | 3624 | XM_001552772 | XP_001552822 | Botryo |
|  |  | *Candida tenuis* | 3702 | XM_006683639 | XP_006683702 | Candida |
|  |  | *Capronia coronata* | 3783 | XM_007727930 | XP_007726120 | Capron |
|  |  | *Cladophialophora yegresii* | 3801 | XM_007756041 | XP_007754231 | Cladoph |
|  |  | *Clavispora lusitaniae* | 2577 | XM_002616342 | XP_002616388 | Clavisp |
|  |  | *Colletotrichum fioriniae* | 3777 | XM_007602979 | XP_007603041 | Collet |
|  |  | *Cordyceps militaris* | 3786 | XM_006671835 | XP_006671898 | Cordyce |
|  |  | *Debaryomyces hansenii* | 3699 | XM_002770548 | XP_002770594 | Debaryo |
|  |  | *Endocarpon pusillum* | 3795 | XM_007803604 | XP_007801795 | Endocar |
|  |  | *Eutypa lata* | 3780 | XM_007797488 | XP_007795679 | Eutypa |
|  |  | *Glarea lozoyensis* | 3792 | XM_008080462 | XP_008078653 | Glarea |
|  |  | *Kazachstania africana* | 3669 | XM_003957484 | XP_003957533 | Kazach |
|  |  | *Kluyveromyces lactis* | 3666 | XM_451784 | XP_451784 | Kluyver |
|  |  | *Lachancea thermotolerans* | 3663 | XM_002555122 | XP_002555168 | Lachan |
|  |  | *Leptosphaeria maculans* | 4083 | XM_003841144 | XP_003841192 | Leptos |
|  |  | *Marssonina brunnea* | 3801 | XM_007292508 | XP_007292570 | Marsson |
|  |  | *Metarhizium acridum* | 3792 | XM_007810612 | XP_007808803 | Metarh |
|  |  | *Meyerozyma guilliermondii* | 3876 | XM_001485054 | XP_001485104 | Meyero |
|  |  | *Millerozyma farinosa* | 3702 | XM_004203277 | XP_004203325 | Millero |
|  |  | *Nectria* *haematococca* | 3786 | XM_003045274 | XP_003045320 | Nectria |
|  |  | *Neofusicoccum parvum* | 3774 | XM_007585445 | XP_007585507 | Neofusic |
|  |  | *Neosartorya fischeri* | 3768 | XM_001262828 | XP_001262829 | Neosar |
|  |  | *Neurospora crassa* | 3018 | XM_952013 | XP_957106 | Neuros |
|  |  | *Penicillium chrysogenum* | 3768 | XM_002568249 | XP_002568295 | Penici |
|  |  | *Pestalotiopsis fici* | 3789 | XM_007830789 | XP_007828980 | Pestalo |
|  |  | *Podospora anserina* | 3837 | XM_001903753 | XP_001903788 | Podosp |
|  |  | *Pseudocercospora fijiensis* | 3747 | XM_007927936 | XP_007926127 | Pseudoce |
|  |  | *Pyrenophora tritici-repentis* | 3627 | XM_001934908 | XP_001934943 | Pyreno |
|  |  | *Saccharomyces cerevisiae* | 3675 | NM_001183570 | NP_014794 | Saccha |
|  |  | *Sclerotinia sclerotiorum* | 3804 | XM_001598746 | XP_001598796 | Sclero |
|  |  | *Sordaria macrospora* | 3825 | XM_003347356 | XP_003347404 | Sordar |
|  |  | *Talaromyces stipitatus* | 3771 | XM_002488267 | XP_002488312 | Talarom |
|  |  | *Togninia minima* | 3795 | XM_007918510 | XP_007916701 | Togni |
|  |  | *Torulaspora delbrueckii* | 3669 | XM_003678856 | XP_003678904 | Torula |
|  |  | *Trichoderma reesei* | 3789 | XM_006966461 | XP_006966523 | Trichode |
|  |  | *Trichophyton rubrum* | 3777 | XM_003233825 | XP_003233873 | Tricho |
|  |  | *Verticillium alfalfae* | 3783 | XM_003006316 | XP_003006362 | Vertici |
|  |  | *Zygosaccharomyces rouxii* | 3675 | XM_002496900 | XP_002496945 | Zygosa |
|  | Basidiomycota | *Agaricus bisporus* | 3663 | XM_007325766 | XP_007325828 | Agaric |
|  |  | *Auricularia delicata* | 3654 | XM_007339757 | XP_007339819 | Auricu |
|  |  | *Coniophora puteana* | 3687 | XM_007765472 | XP_007763662 | Conioph |
|  |  | *Coprinopsis cinerea* | 3669 | XM_001829088 | XP_001829140 | Coprino |
|  |  | *Dichomitus squalens* | 3669 | XM_007360267 | XP_007360329 | Dicho |
|  |  | *Fomitiporia mediterranea* | 3825 | XM_007265144 | XP_007265206 | Fomitip |
|  |  | *Gloeophyllum trabeum* | 3657 | XM_007862081 | XP_007860272 | Gloeoph |
|  |  | *Melampsora larici-populina* | 3774 | XM_007407916 | XP_007407978 | Melamp |
|  |  | *Moniliophthora roreri* | 3690 | XM_007846986 | XP_007845177 | Monilio |
|  |  | *Phanerochaete carnosa* | 3714 | XM_007390692 | XP_007390754 | Phanero |
|  |  | *Pseudozyma flocculosa* | 3753 | XM_007880762 | XP_007878953 | Psudoz |
|  |  | *Puccinia graminis* | 3396 | XM_003321826 | XP_003321874 | Puccinia |
|  |  | *Punctularia strigosozonata* | 3744 | XM_007378843 | XP_007378905 | Punctu |
|  |  | *Schizophyllum commune* | 3615 | XM_003038777 | XP_003038823 | Schizo |
|  |  | *Serpula lacrymans* | 3630 | XM_007313109 | XP_007313171 | Serpu |
|  |  | *Stereum hirsutum* | 3693 | XM_007298209 | XP_007298271 | Stereum |
|  |  | *Trametes versicolor* | 3696 | XM_008033763 | XP_008031954 | Tramet |
|  |  | *Tremella mesenterica* | 3645 | XM_007002026 | XP_007002088 | Treme |
|  |  | *Ustilago maydis* | 2661 | AAS67525 | AAS67525 | Ustilago |
|  |  | *Wallemia sebi* | 3597 | XM_006957510 | XP_006957572 | Wallem |
|  | Microsporidia | *Nosema ceranae* | 3432 | XM_002995943 | XP_002995989 | Nosema |
| Archaea ** | | *Halorhabdus utahensis* | 1827 | AB477175 | BAH80364 | Halorh |
|  |  | *Methanosarcina barkeri* | 1815 | CP000099 | AAZ72556 | Methano |
| Bacteria ** | | *Cronobacter sakazakii* | 4029 | CP006731 | AHB72209 | Cronob |
|  |  | *Geobacter sulfurreducens* | 4113 | CP002031 | ADI85618 | Geoba |
|  |  | *Gloeobacter violaceus* | 3339 | BA000045 | BAC90224 | Gloeob |
|  |  | *Spirosoma linguale* | 3816 | CP001769 | ADB40457 | Spiros |

* DNA sequences are not available

** Outgroups
