# Supplementary material for: Evolutionary History of the Photolyase/Cryptochrome Superfamily in Eukaryotes
Source: PLoS One. 2015 Sep 9;10(9):e0135940. doi: 10.1371/journal.pone.0135940 (PMC4564169; doi:10.1371/journal.pone.0135940)
Supplement: S3 Table — Mean ω was obtained using local MG94 model (no site-to-site variation). Values of ω+ and weight ω+ reflect the strength of selection and the proportion of the total branch length of positive selection. Estimates of the uncorrected p-value were generated by the mixture of distributions, corrected p was the probability obtained after Holm’s correction for multiple testing. Branches with mean ω > 1 are shaded. Infinite mean ω for branches may be resulted by lack of synonymous substitution. As a result, they were not considered to be positively selected branches. (DOCX) [file pone.0135940.s005.docx]

S3 Table. Results of episodic diversifying selection test by REL

| Branch ^a^ | Mean ω | Rate Class | ω^+^ | Weight ω^+^ | *p* | Corrected *p* |
| --- | --- | --- | --- | --- | --- | --- |
| **Node6** | **2.077** | **2** | **infinity** | **0.304** | **1.000** | **1.000** |
| Node14 | 0.237 | 2 | 9.823 | 0.193 | 1.000 | 1.000 |
| Node13 | infinity | 2 | infinity | 0.088 | 1.000 | 1.000 |
| Node11 | 0.578 | 1 | infinity | 1.000 | 1.000 | 1.000 |
| Node31 | 0.216 | 2 | infinity | 0.055 | 1.000 | 1.000 |
| Node39 | 0.185 | 2 | 1.217 | 0.020 | 1.000 | 1.000 |
| Node48 | 0.722 | 2 | 92.223 | 0.047 | 1.000 | 1.000 |
| Node44 | 0.275 | 2 | infinity | 0.053 | 1.000 | 1.000 |
| Node28 | infinity | 1 | infinity | 1.000 | 1.000 | 1.000 |
| Node63 | 0.110 | 3 | infinity | 0.323 | 1.000 | 1.000 |
| Node61 | 0.248 | 2 | 1.224 | 0.033 | 1.000 | 1.000 |
| Node59 | 0.778 | 2 | infinity | 0.034 | 1.000 | 1.000 |
| Node53 | 0.202 | 2 | infinity | 0.006 | 1.000 | 1.000 |
| Node51 | infinity | 2 | 2.074 | 0.031 | 1.000 | 1.000 |
| **Node70** | **2.841** | **2** | **infinity** | **0.037** | **1.000** | **1.000** |
| **Node68** | **1.031** | **2** | **infinity** | **0.128** | **1.000** | **1.000** |
| Node26 | infinity | 1 | infinity | 1.000 | 1.000 | 1.000 |
| Node10 | infinity | 2 | infinity | 0.029 | 1.000 | 1.000 |
| Node81 | infinity | 2 | 1.540 | 0.017 | 1.000 | 1.000 |
| Node79 | 0.722 | 1 | infinity | 1.000 | 1.000 | 1.000 |
| Node91 | 0.077 | 2 | infinity | 0.027 | 1.000 | 1.000 |
| Node103 | infinity | 1 | infinity | 1.000 | 1.000 | 1.000 |
| **Node99** | **1.433** | **2** | **5230.128** | **0.043** | **1.000** | **1.000** |
| Node89 | 0.032 | 2 | infinity | 0.011 | 1.000 | 1.000 |
| Node108 | 0.047 | 2 | 20.658 | 0.029 | 1.000 | 1.000 |
| Node106 | infinity | 1 | infinity | 1.000 | 1.000 | 1.000 |
| Node88 | 0.593 | 2 | 91.552 | 0.034 | 1.000 | 1.000 |
| **Node111** | **1.320** | **2** | **infinity** | **0.022** | **1.000** | **1.000** |
| Node87 | infinity | 2 | 67.736 | 0.019 | 1.000 | 1.000 |
| Node116 | 0.310 | 2 | infinity | 0.094 | 1.000 | 1.000 |
| Node114 | infinity | 2 | infinity | 0.052 | 1.000 | 1.000 |
| Node86 | infinity | 2 | 3.492 | 0.010 | 1.000 | 1.000 |
| Node125 | 0.069 | 2 | 1.004 | 0.055 | 1.000 | 1.000 |
| **Node132** | **7.178** | **1** | **1.088** | **1.000** | **1.000** | **1.000** |
| Node128 | infinity | 1 | infinity | 1.000 | 1.000 | 1.000 |
| Node124 | 0.727 | 2 | infinity | 0.062 | 1.000 | 1.000 |
| **Node135** | **5.945** | **2** | **infinity** | **0.040** | **1.000** | **1.000** |
| Node121 | 0.087 | 2 | infinity | 0.075 | 1.000 | 1.000 |
| Node73 | infinity | 2 | infinity | 0.095 | 1.000 | 1.000 |
| Node9 | 0.863 | 2 | infinity | 0.163 | 1.000 | 1.000 |
| Node3 | infinity | 2 | infinity | 0.224 | 1.000 | 1.000 |
| Node158 | infinity | 2 | infinity | 0.060 | 1.000 | 1.000 |
| Node163 | 0.107 | 2 | 250.602 | 0.073 | 1.000 | 1.000 |
| Node161 | 0.099 | 2 | infinity | 0.057 | 1.000 | 1.000 |
| Node157 | infinity | 2 | infinity | 0.161 | 1.000 | 1.000 |
| Node155 | infinity | 2 | infinity | 0.052 | 1.000 | 1.000 |
| Node175 | infinity | 1 | infinity | 1.000 | 1.000 | 1.000 |
| Node153 | infinity | 2 | 3.101 | 0.312 | 1.000 | 1.000 |
| Node184 | 0.122 | 1 | 1.823 | 1.000 | 1.000 | 1.000 |
| Node182 | infinity | 1 | infinity | 1.000 | 1.000 | 1.000 |
| Node181 | infinity | 2 | infinity | 0.269 | 1.000 | 1.000 |
| Node199 | infinity | 2 | infinity | 0.095 | 1.000 | 1.000 |
| Node198 | infinity | 1 | infinity | 1.000 | 1.000 | 1.000 |
| Node196 | infinity | 2 | infinity | 0.139 | 1.000 | 1.000 |
| Node180 | 0.006 | 2 | 87.865 | 0.032 | 1.000 | 1.000 |
| Node230 | 0.000 | 2 | 587.585 | 0.057 | 1.000 | 1.000 |
| Node232 | infinity | 1 | infinity | 1.000 | 1.000 | 1.000 |
| Node229 | infinity | 2 | infinity | 0.062 | 1.000 | 1.000 |
| Node227 | 0.186 | 2 | infinity | 0.079 | 1.000 | 1.000 |
| Node239 | 0.129 | 2 | 9.554 | 0.028 | 1.000 | 1.000 |
| Node238 | 0.053 | 2 | infinity | 0.020 | 1.000 | 1.000 |
| Node236 | infinity | 2 | 1.845 | 0.010 | 1.000 | 1.000 |
| Node224 | infinity | 1 | infinity | 1.000 | 1.000 | 1.000 |
| Node254 | 0.080 | 2 | infinity | 0.042 | 1.000 | 1.000 |
| Node252 | infinity | 2 | infinity | 0.065 | 1.000 | 1.000 |
| Node250 | infinity | 2 | 2.573 | 0.090 | 1.000 | 1.000 |
| Node257 | infinity | 1 | infinity | 1.000 | 1.000 | 1.000 |
| Node209 | 0.225 | 2 | infinity | 0.078 | 1.000 | 1.000 |
| Node264 | infinity | 2 | infinity | 0.098 | 1.000 | 1.000 |
| Node262 | infinity | 2 | infinity | 0.020 | 1.000 | 1.000 |
| Node208 | infinity | 2 | infinity | 0.076 | 1.000 | 1.000 |
| Node273 | 0.041 | 2 | infinity | 0.053 | 1.000 | 1.000 |
| Node269 | infinity | 1 | infinity | 1.000 | 1.000 | 1.000 |
| Node267 | 0.496 | 2 | 3.923 | 0.255 | 1.000 | 1.000 |
| Node207 | infinity | 1 | infinity | 1.000 | 1.000 | 1.000 |
| Node205 | 0.824 | 1 | infinity | 1.000 | 1.000 | 1.000 |
| Node289 | 0.000 | 2 | infinity | 0.175 | 1.000 | 1.000 |
| Node283 | 0.000 | 2 | infinity | 0.046 | 1.000 | 1.000 |
| Node291 | 0.000 | 2 | 1.179 | 0.099 | 1.000 | 1.000 |
| Node296 | infinity | 1 | infinity | 1.000 | 1.000 | 1.000 |
| Node299 | infinity | 2 | infinity | 0.053 | 1.000 | 1.000 |
| Node311 | infinity | 2 | infinity | 0.083 | 1.000 | 1.000 |
| Node314 | infinity | 2 | infinity | 0.024 | 1.000 | 1.000 |
| Node310 | infinity | 2 | 2.245 | 0.057 | 1.000 | 1.000 |
| Node288 | infinity | 1 | infinity | 1.000 | 1.000 | 1.000 |
| Node282 | infinity | 2 | infinity | 0.055 | 1.000 | 1.000 |
| Node178 | infinity | 2 | infinity | 0.089 | 1.000 | 1.000 |
| Node152 | 0.000 | 2 | infinity | 0.136 | 1.000 | 1.000 |
| Node150 | 0.000 | 2 | 4.576 | 0.132 | 1.000 | 1.000 |
| Node140 | infinity | 2 | infinity | 0.236 | 1.000 | 1.000 |
| Node329 | 0.000 | 2 | infinity | 0.109 | 1.000 | 1.000 |
| **Node340** | **1.443** | **2** | **99.527** | **0.082** | **1.000** | **1.000** |
| Node345 | 0.158 | 3 | 3.001 | 0.060 | 1.000 | 1.000 |
| Node354 | 0.077 | 2 | 84.013 | 0.009 | 1.000 | 1.000 |
| Node350 | 0.319 | 2 | infinity | 0.077 | 1.000 | 1.000 |
| Node359 | 0.171 | 3 | 24.975 | 0.064 | 1.000 | 1.000 |
| Node349 | infinity | 2 | 95.407 | 0.027 | 1.000 | 1.000 |
| **Node367** | **6.202** | **2** | **infinity** | **0.054** | **1.000** | **1.000** |
| Node379 | infinity | 2 | infinity | 0.121 | 1.000 | 1.000 |
| Node377 | infinity | 1 | 1.131 | 1.000 | 1.000 | 1.000 |
| Node394 | infinity | 2 | infinity | 0.068 | 1.000 | 1.000 |
| Node417 | 0.089 | 2 | infinity | 0.101 | 1.000 | 1.000 |
| Node415 | infinity | 2 | infinity | 0.072 | 1.000 | 1.000 |
| Node423 | 0.045 | 2 | infinity | 0.043 | 1.000 | 1.000 |
| Node433 | infinity | 2 | 90.522 | 0.037 | 1.000 | 1.000 |
| Node440 | infinity | 2 | 50.781 | 0.056 | 1.000 | 1.000 |
| Node428 | 0.712 | 2 | infinity | 0.132 | 1.000 | 1.000 |
| Node392 | infinity | 1 | infinity | 1.000 | 1.000 | 1.000 |
| Node390 | 0.040 | 2 | infinity | 0.083 | 1.000 | 1.000 |
| Node387 | 0.000 | 2 | infinity | 0.040 | 1.000 | 1.000 |
| Node385 | infinity | 2 | infinity | 0.098 | 1.000 | 1.000 |
| Node470 | 0.943 | 2 | 13.358 | 0.113 | 1.000 | 1.000 |
| Node468 | 0.199 | 2 | infinity | 0.025 | 1.000 | 1.000 |
| Node473 | 0.854 | 2 | infinity | 0.134 | 1.000 | 1.000 |
| Node467 | 0.438 | 2 | infinity | 0.109 | 1.000 | 1.000 |
| Node478 | infinity | 1 | infinity | 1.000 | 1.000 | 1.000 |
| Node476 | 0.178 | 2 | infinity | 0.124 | 1.000 | 1.000 |
| Node466 | infinity | 1 | infinity | 1.000 | 1.000 | 1.000 |
| Node465 | infinity | 2 | infinity | 0.127 | 1.000 | 1.000 |
| Node464 | infinity | 2 | infinity | 0.071 | 1.000 | 1.000 |
| Node488 | 0.000 | 2 | infinity | 0.209 | 1.000 | 1.000 |
| Node512 | infinity | 2 | 75.216 | 0.031 | 1.000 | 1.000 |
| Node510 | 0.113 | 2 | infinity | 0.061 | 1.000 | 1.000 |
| **Node508** | **1.082** | **2** | **87.502** | **0.038** | **1.000** | **1.000** |
| **Node519** | **2.481** | **2** | **83.830** | **0.029** | **1.000** | **1.000** |
| Node536 | 0.348 | 2 | 25.625 | 0.031 | 1.000 | 1.000 |
| Node499 | 0.116 | 2 | infinity | 0.156 | 1.000 | 1.000 |
| Node491 | 0.000 | 2 | infinity | 0.158 | 1.000 | 1.000 |
| Node463 | infinity | 1 | infinity | 1.000 | 1.000 | 1.000 |
| Node573 | 0.681 | 2 | infinity | 0.020 | 1.000 | 1.000 |
| **Node571** | **5.777** | **2** | **infinity** | **0.037** | **1.000** | **1.000** |
| Node563 | 0.484 | 2 | 88.925 | 0.039 | 1.000 | 1.000 |
| Node553 | 0.070 | 2 | infinity | 0.049 | 1.000 | 1.000 |
| Node583 | infinity | 1 | infinity | 1.000 | 1.000 | 1.000 |
| Node581 | 0.000 | 2 | 1.698 | 0.014 | 1.000 | 1.000 |
| Node590 | 0.484 | 2 | 7.480 | 0.045 | 1.000 | 1.000 |
| **Node595** | **1.180** | **2** | **1.250** | **0.030** | **1.000** | **1.000** |
| Node593 | 0.156 | 2 | infinity | 0.051 | 1.000 | 1.000 |
| Node589 | infinity | 1 | infinity | 1.000 | 1.000 | 1.000 |
| Node599 | 0.239 | 2 | 2.255 | 0.052 | 1.000 | 1.000 |
| Node602 | 0.286 | 2 | infinity | 0.036 | 1.000 | 1.000 |
| Node588 | 0.171 | 2 | 4.372 | 0.060 | 1.000 | 1.000 |
| Node586 | 0.116 | 2 | infinity | 0.024 | 1.000 | 1.000 |
| Node462 | infinity | 2 | infinity | 0.046 | 1.000 | 1.000 |
| Node384 | infinity | 1 | infinity | 1.000 | 1.000 | 1.000 |
| **Node382** | **1.713** | **2** | **infinity** | **0.046** | **1.000** | **1.000** |
| Node376 | infinity | 2 | infinity | 0.085 | 1.000 | 1.000 |
| Node320 | infinity | 2 | infinity | 0.205 | 1.000 | 1.000 |
| Node622 | 0.021 | 2 | infinity | 0.011 | 1.000 | 1.000 |
| Node621 | 0.650 | 2 | 1.606 | 0.090 | 1.000 | 1.000 |
| Node632 | 0.115 | 2 | infinity | 0.036 | 1.000 | 1.000 |
| Node620 | 0.537 | 2 | 3.222 | 0.120 | 1.000 | 1.000 |
| Node637 | infinity | 2 | 10.854 | 0.027 | 1.000 | 1.000 |
| Node635 | 0.328 | 2 | 4.116 | 0.109 | 1.000 | 1.000 |
| Node619 | 0.177 | 2 | infinity | 0.062 | 1.000 | 1.000 |
| Node652 | 0.169 | 2 | infinity | 0.033 | 1.000 | 1.000 |
| Node651 | infinity | 2 | 2.374 | 0.020 | 1.000 | 1.000 |
| Node648 | infinity | 2 | infinity | 0.050 | 1.000 | 1.000 |
| Node640 | 0.199 | 2 | infinity | 0.058 | 1.000 | 1.000 |
| Node616 | infinity | 2 | infinity | 0.034 | 1.000 | 1.000 |
| Node662 | 0.087 | 2 | infinity | 0.045 | 1.000 | 1.000 |
| Node665 | 0.217 | 2 | infinity | 0.169 | 1.000 | 1.000 |
| Node661 | 0.748 | 2 | infinity | 0.057 | 1.000 | 1.000 |
| Node615 | infinity | 2 | infinity | 0.084 | 1.000 | 1.000 |
| Node676 | 0.000 | 2 | infinity | 0.251 | 1.000 | 1.000 |
| Node668 | infinity | 2 | 3.900 | 0.070 | 1.000 | 1.000 |
| Node612 | 0.000 | 2 | infinity | 0.146 | 1.000 | 1.000 |
| Node610 | 0.000 | 2 | infinity | 0.101 | 1.000 | 1.000 |
| Node687 | 0.676 | 2 | infinity | 0.155 | 1.000 | 1.000 |
| Node685 | infinity | 2 | infinity | 0.088 | 1.000 | 1.000 |
| Node695 | 0.122 | 2 | 1.156 | 0.124 | 1.000 | 1.000 |
| Node693 | infinity | 2 | infinity | 0.061 | 1.000 | 1.000 |
| Node708 | 0.147 | 2 | infinity | 0.017 | 1.000 | 1.000 |
| Node713 | 0.092 | 2 | infinity | 0.089 | 1.000 | 1.000 |
| Node701 | 0.353 | 1 | 1.391 | 1.000 | 1.000 | 1.000 |
| Node722 | 0.103 | 2 | 3.059 | 0.052 | 1.000 | 1.000 |
| Node740 | 0.092 | 2 | infinity | 0.075 | 1.000 | 1.000 |
| Node736 | 0.067 | 2 | 1.022 | 0.115 | 1.000 | 1.000 |
| Node690 | 0.095 | 2 | infinity | 0.068 | 1.000 | 1.000 |
| Node684 | 0.195 | 2 | infinity | 0.044 | 1.000 | 1.000 |
| Node682 | infinity | 2 | infinity | 0.055 | 1.000 | 1.000 |
| Node680 | infinity | 1 | 10.200 | 1.000 | 1.000 | 1.000 |
| Node747 | 0.000 | 2 | infinity | 0.061 | 1.000 | 1.000 |
| Node762 | 0.000 | 2 | infinity | 0.147 | 1.000 | 1.000 |
| Node769 | 0.000 | 2 | infinity | 0.019 | 1.000 | 1.000 |
| Node761 | 0.000 | 2 | infinity | 0.105 | 1.000 | 1.000 |
| Node759 | 0.000 | 2 | infinity | 0.102 | 1.000 | 1.000 |
| Node757 | 0.000 | 2 | infinity | 0.072 | 1.000 | 1.000 |
| Node775 | infinity | 1 | infinity | 1.000 | 1.000 | 1.000 |
| Node789 | infinity | 1 | infinity | 1.000 | 1.000 | 1.000 |
| Node801 | 0.098 | 2 | infinity | 0.053 | 1.000 | 1.000 |
| Node795 | infinity | 2 | 1.864 | 0.012 | 1.000 | 1.000 |
| Node779 | 0.056 | 2 | infinity | 0.023 | 1.000 | 1.000 |
| Node808 | infinity | 1 | infinity | 1.000 | 1.000 | 1.000 |
| Node804 | 0.142 | 2 | infinity | 0.050 | 1.000 | 1.000 |
| Node774 | 0.522 | 2 | infinity | 0.081 | 1.000 | 1.000 |
| Node772 | 0.174 | 2 | infinity | 0.027 | 1.000 | 1.000 |
| Node756 | infinity | 1 | infinity | 1.000 | 1.000 | 1.000 |
| Node754 | infinity | 2 | infinity | 0.050 | 1.000 | 1.000 |
| Node823 | infinity | 1 | infinity | 1.000 | 1.000 | 1.000 |
| Node828 | infinity | 1 | infinity | 1.000 | 1.000 | 1.000 |
| Node857 | infinity | 2 | infinity | 0.105 | 1.000 | 1.000 |
| Node856 | infinity | 2 | infinity | 0.045 | 1.000 | 1.000 |
| Node852 | infinity | 2 | infinity | 0.111 | 1.000 | 1.000 |
| Node894 | 0.021 | 2 | infinity | 0.016 | 1.000 | 1.000 |
| Node892 | 0.000 | 2 | 103.354 | 0.063 | 1.000 | 1.000 |
| Node874 | 0.003 | 2 | infinity | 0.074 | 1.000 | 1.000 |
| **Node916** | **1.167** | **1** | **1.305** | **1.000** | **1.000** | **1.000** |
| Node1048 | infinity | 1 | infinity | 1.000 | 1.000 | 1.000 |
| **Node1054** | **2.417** | **2** | **97.808** | **0.021** | **1.000** | **1.000** |
| Node1061 | 0.037 | 2 | infinity | 0.030 | 1.000 | 1.000 |
| Node1059 | 0.420 | 2 | 5.141 | 0.021 | 1.000 | 1.000 |
| Node1086 | 0.054 | 2 | 2.458 | 0.072 | 1.000 | 1.000 |
| Node1084 | infinity | 1 | infinity | 1.000 | 1.000 | 1.000 |
| Node1082 | infinity | 1 | infinity | 1.000 | 1.000 | 1.000 |
| Node1081 | 0.040 | 2 | infinity | 0.003 | 1.000 | 1.000 |
| Node1079 | 0.040 | 2 | infinity | 0.119 | 1.000 | 1.000 |
| Node1075 | 0.192 | 1 | infinity | 1.000 | 1.000 | 1.000 |
| Node1106 | infinity | 1 | infinity | 1.000 | 1.000 | 1.000 |
| Node1112 | 0.028 | 2 | infinity | 0.015 | 1.000 | 1.000 |
| **Node1074** | **1.056** | **1** | **infinity** | **1.000** | **1.000** | **1.000** |
| Node1069 | infinity | 1 | infinity | 1.000 | 1.000 | 1.000 |
| Node1125 | infinity | 1 | infinity | 1.000 | 1.000 | 1.000 |
| Node1124 | infinity | 1 | infinity | 1.000 | 1.000 | 1.000 |
| Node1144 | 0.391 | 2 | infinity | 0.020 | 1.000 | 1.000 |
| Node1118 | 0.238 | 1 | infinity | 1.000 | 1.000 | 1.000 |
| Node1170 | infinity | 1 | infinity | 1.000 | 1.000 | 1.000 |
| Node1162 | infinity | 1 | 7.075 | 1.000 | 1.000 | 1.000 |
| Node1320 | infinity | 2 | infinity | 0.007 | 1.000 | 1.000 |
| Node1319 | infinity | 2 | infinity | 0.006 | 1.000 | 1.000 |
| Node1149 | 0.151 | 2 | infinity | 0.023 | 1.000 | 1.000 |
| Node901 | 0.406 | 2 | infinity | 0.071 | 1.000 | 1.000 |
| Node899 | infinity | 1 | infinity | 1.000 | 1.000 | 1.000 |
| Node873 | infinity | 2 | infinity | 0.038 | 1.000 | 1.000 |
| Node871 | infinity | 1 | infinity | 1.000 | 1.000 | 1.000 |
| Node867 | infinity | 2 | infinity | 0.097 | 1.000 | 1.000 |
| Node843 | infinity | 2 | infinity | 0.104 | 1.000 | 1.000 |
| Node813 | infinity | 2 | infinity | 0.129 | 1.000 | 1.000 |
| Node753 | infinity | 2 | infinity | 0.095 | 1.000 | 1.000 |
| Node745 | 0.177 | 2 | infinity | 0.083 | 1.000 | 1.000 |
| Node679 | 0.750 | 2 | infinity | 0.100 | 1.000 | 1.000 |
| Node607 | infinity | 2 | infinity | 0.164 | 1.000 | 1.000 |
| **Node605** | **2.863** | **2** | **infinity** | **0.138** | **1.000** | **1.000** |
| **Node319** | **6.495** | **2** | **infinity** | **0.139** | **1.000** | **1.000** |
| Node1353 | 0.000 | 2 | infinity | 0.173 | 1.000 | 1.000 |
| Node1351 | 0.000 | 2 | infinity | 0.099 | 1.000 | 1.000 |
| Node1370 | 0.004 | 2 | infinity | 0.047 | 1.000 | 1.000 |
| Node1368 | infinity | 2 | infinity | 0.048 | 1.000 | 1.000 |
| Node1376 | 0.000 | 2 | infinity | 1.834 | 1.000 | 1.000 |
| Node1409 | 0.117 | 2 | infinity | 0.059 | 1.000 | 1.000 |
| Node1405 | 0.343 | 1 | infinity | 1.000 | 1.000 | 1.000 |
| Node1418 | 0.535 | 2 | infinity | 0.120 | 1.000 | 1.000 |
| Node1414 | 0.098 | 3 | infinity | 0.038 | 1.000 | 1.000 |
| Node1423 | infinity | 1 | infinity | 1.000 | 1.000 | 1.000 |
| Node1387 | infinity | 2 | infinity | 0.104 | 1.000 | 1.000 |
| Node1373 | infinity | 2 | infinity | 0.160 | 1.000 | 1.000 |
| Node1440 | 0.000 | 2 | infinity | 0.066 | 1.000 | 1.000 |
| Node1434 | 0.000 | 2 | infinity | 0.066 | 1.000 | 1.000 |
| Node1449 | 0.184 | 3 | infinity | 0.056 | 1.000 | 1.000 |
| Node1447 | infinity | 2 | 84.315 | 0.054 | 1.000 | 1.000 |
| Node1445 | 0.000 | 1 | infinity | 1.000 | 1.000 | 1.000 |
| Node1433 | 0.000 | 2 | infinity | 0.099 | 1.000 | 1.000 |
| Node1465 | 0.311 | 2 | 2.326 | 0.019 | 1.000 | 1.000 |
| Node1476 | infinity | 1 | infinity | 1.000 | 1.000 | 1.000 |
| Node1487 | 0.644 | 2 | infinity | 0.025 | 1.000 | 1.000 |
| **Node1502** | **1.232** | **2** | **550.955** | **0.056** | **1.000** | **1.000** |
| Node1494 | 0.330 | 2 | 1.114 | 0.186 | 1.000 | 1.000 |
| Node1366 | 0.000 | 2 | infinity | 0.062 | 1.000 | 1.000 |
| Node1360 | 0.000 | 2 | infinity | 0.065 | 1.000 | 1.000 |
| Node1350 | 0.000 | 2 | infinity | 0.055 | 1.000 | 1.000 |

Mean ω was obtained using local MG94 model (no site-to-site variation). Values of ω^+^ and weight ω^+^ reflect the strength of selection and the proportion of the total branch length of positive selection. Estimates of the uncorrected *p*-value were generated by the mixture of distributions, corrected *p* was the probability obtained after Holm’s correction for multiple testing. Branches with mean ω > 1 are in bold. Infinite mean ω for branches may be resulted by lack of synonymous substitution. As a result, they were not considered to be positively selected branches.
